# Discovery of thiacyanine dyes as a new class of potent coronavirus inhibitors that suppress viral RNA synthesis

**DOI:** 10.1016/j.jbc.2025.110547

**Published:** 2025-08-05

**Authors:** Tianyi Zhang, Valerie Altouma, Joshua A. Sommers, Adaira J. Dumm, Debbie Kennedy, Hongjie Xia, Mo Yang, Christopher R. Fullenkamp, Shar-yin N. Huang, Yutong Xue, Shuaikun Su, Weiping Shen, Caitlin E. Haren, Tomasz Kulikowicz, Yves Pommier, John S. Schneekloth, Mariano A. Garcia-Blanco, Robert M. Brosh, Weidong Wang

**Affiliations:** 1 Lab of Genetics and Genomics, National Institute on Aging, NIH, Baltimore, Maryland, USA; 2 Translational Gerontology Branch, National Institute on Aging, NIH, Baltimore, Maryland, USA; 3 Department of Microbiology, Immunology and Cancer Biology, University of Virginia, Charlottesville, Virginia, USA; 4 Department of Biochemistry and Molecular Biology, University of Texas Medical Branch, Galveston, Texas, USA; 5 Department of Microbiology & Immunology, University of Texas Medical Branch, Galveston, Texas, USA; 6 Chemical Biology Laboratory, Center for Cancer Research, National Cancer Institute, Frederick, Maryland, USA; 7 Developmental Therapeutics Branch and Laboratory of Molecular Pharmacology, Center for Cancer Research, National Cancer Institute, NIH, Bethesda, Maryland, USA

**Keywords:** topoisomerase, antiviral drugs, TOP3B, TDRD3, coronavirus, MHV, SARS

## Abstract

Topoisomerase poisons are clinically used anticancer drugs that can induce DNA cleavage complexes to block replication. TOP3B is the only topoisomerase that can catalyze topological changes on either DNA or RNA and induce cleavage complexes on both nucleic acids. We proposed that TOP3B poisons may inhibit coronavirus RNA genome replication and tested this hypothesis by using mouse hepatitis coronavirus (MHV). We found that one of the two types of reported TOP3B poisons, thiacyanine dyes, possess potent inhibitory activities for MHV. Interestingly, the antiviral activity of the thiacyanine dyes is unaltered in *Top3b*-KO cells, suggesting that these dyes inhibit viral replication independent of TOP3B. Subsequent screening revealed that multiple members of the thiacyanine dye family have antiviral activity comparable to or stronger than remdesivir, the U.S. Food and Drug Administration (FDA)-approved drug for coronavirus, in an MHV-infected cell line model. One thiacyanine dye (NSC93472) significantly inhibits MHV replication in mouse lungs, showing its potential as an anticoronavirus drug. Mechanistic studies showed that NSC93472 preferentially binds two RNA fragments derived from SARS-CoV-2 genome over random ssRNA, interferes with assembly of an elongation-competent complex between the viral RNA-dependent RNA polymerase and the RNA template, and inhibits the RNA synthesis mediated by the RNA-dependent RNA polymerase. Moreover, NSC93472 can inhibit RNA synthesis by the reverse transcriptase of Moloney murine leukemia virus. Our studies demonstrate that thiacyanine dyes represent a new family of coronavirus inhibitors and suggest that TOP3B poisons and anti-RNA virus drugs share common characteristics in RNA binding and inhibition of enzymatic reactions on RNA.

Pandemics caused by RNA viruses, including the recent severe acute respiratory syndrome coronavirus 2 (SARS-CoV-2), have resulted in world-wide catastrophes, causing severe disease and death in millions of people, and disrupting daily lives of many more (1, 2). The severity of these past pandemics requires the development of new antiviral strategies to combat potential future pandemics caused by the same or different RNA viruses. Here, we tested the anticoronavirus activities of TOP3B poisons discovered recently (3).

TOP3B is one of six topoisomerases in animals (others are TOP1, TOP1MT, TOP2A, TOP2B, and TOP3A). These enzymes have a unique capacity to catalyze strand passage reactions: they can induce a transient single- or double-strand break, allow the other unbroken strand(s) to pass through the break to release topological stress, and then reseal the broken ends (4). All topoisomerases produce an intermediate structure, called DNA-cleavage complex, consisting of broken ends of DNA covalently linked to a tyrosine residue at the catalytic site of topoisomerases. This complex has been specifically targeted for the development of therapeutic compounds, which can trap the complex and thus prevent religation of broken DNA. These compounds, referred to as topoisomerase “poisons,” not only inhibit activity of topoisomerases but also cause DNA damage (containing DNA breaks and DNA-protein crosslinks), leading to inhibition of essential processes on DNA, such as replication and transcription (Fig. 1*A* left). To date, topoisomerase poisons have been successfully used in anticancer, antiinflammation, and antibiotic therapies (5, 6).

**Figure 1:**
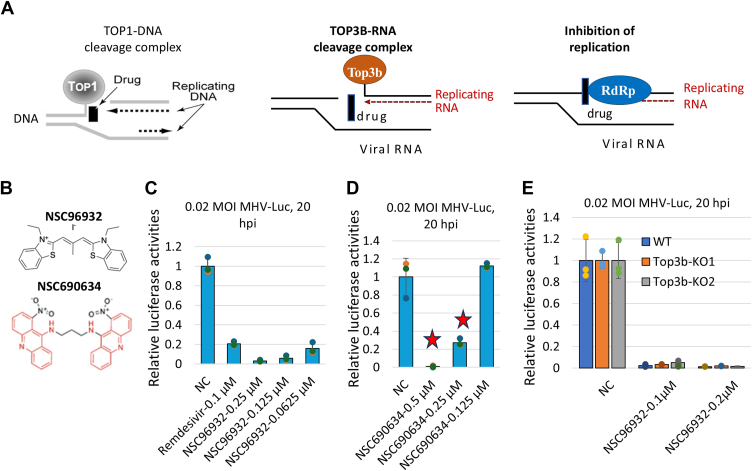
**Thiacyanine dye NSC96932 is a TOP3B poison and shows potent inhibition on coronavirus replication in a TOP3B-independent manner.***A*, schematics of DNA replication inhibited by a TOP1 poison (*left*) and potential functions of a TOP3B poison in inhibition of viral RNA replication by forming TOP3B cleavage complex (*middle*) or inhibiting viral RNA replication enzyme RdRp in a TOP3b-independent manner (*right*). *B*, structures of thiacyanine dye (NSC96932) and bisacridine (NSC690634) represent two classes of TOP3B poisons. *C*, inhibition effects of MHV-Luc activity by NSC96932 at different concentrations. The compound is added into 17Cl-1 cells infected by 0.02 MOI MHV-Luc. The luciferase activities are measured at 20 h post infection (hpi) to monitor MHV-Luc levels. DMSO (0.1%) is used as a negative control, same as below. *D*, inhibition effects of MHV-Luc replication by NSC690634 at different concentrations. *Red stars* indicate that the host cells were detached from plates at these concentrations, indicating high toxicity to the cells. *E*, inhibition effects of MHV-Luc replication in WT and *Top3b*-KO 17Cl-1 cells by NSC96932 at two concentrations. DMSO, dimethyl sulfoxide; MHV, mouse hepatitis coronavirus; MHV-Luc, MHV-Luciferase; MOI, multiplicity of infection; RdRp, RNA-dependent RNA polymerase.

TOP3B is the only topoisomerase in animals that contains an RNA-binding domain and can catalyze strand passage reactions on either DNA or RNA (7, 8, 9). Increasing evidence suggests that TOP3B, and its complex with TDRD3, can function in RNA-based cellular processes, including mRNA translation and turnover (7, 10). Notably, TOP3B has been proposed as an antiviral target, based on evidence that its inactivation reduces replication of multiple positive-sense RNA viruses, including Dengue, Zika, and SARS-Cov2 (11). However, our subsequent analysis showed that Top3b is largely dispensable for replication of mouse hepatitis coronavirus (MHV) (12). Nevertheless, TOP3B poisons may have anticoronavirus activities by the following two mechanisms. First, TOP3B poisons may act in a mechanism like that of DNA-topoisomerase poisons by inducing TOP3B-RNA cleavage complexes on viral RNA to block replication of the viral genome (Fig. 1*A*, middle). Alternatively, some DNA topoisomerase poisons can bind DNA and/or DNA/protein interfaces to desynchronize macromolecular machines that normally function in concert on DNA (13), and it is therefore conceivable that TOP3B poisons may act through another mechanism: binding RNA and/or RNA/protein interface to interfere with enzymes including not only TOP3B but also viral RNA replication machinery (Fig. 1*A*, right).

Two families of TOP3B poisons, bisacridine and thiacyanine, have been discovered by a cell-based screen and they prefer to induce RNA- rather than DNA–cleavage complex (3). Here, we show that a representative member of thiacyanine dyes indeed has potent antiviral activity for mouse coronavirus MHV. Subsequent screening reveals that multiple members of this family have antiviral activity similar to or stronger than remdesivir. Interestingly, these thiacyanines can inhibit viral replication in cells deleted of TOP3B, indicating that their antiviral activity is independent of TOP3B. We further showed that the most potent thiacyanine dye NSC93472 preferentially binds two RNA fragments derived from SARS-CoV-2 genome over a random ssRNA, interfere with assembly of an elongation-competent complex between SARS-CoV-2 RNA-dependent RNA polymerase (RdRp) and RNA template, and inhibit RNA synthesis mediated by the viral replication machinery *in vitro*. Our data demonstrate that thiacyanines can bind RNA and have two independent activities: poisoning of TOP3B to induce RNA damage and inhibition of viral replication by targeting enzymes that replicate viral RNA.

## Results

### A TOP3B poison NSC96932 shows potent antiviral activity against coronavirus MHV

We investigated whether the two known TOP3B poisons can affect replication of an RNA virus, mouse coronavirus MHV, using the same cell line and mouse models described previously (12). Briefly, we used an MHV-Luciferase (MHV-Luc) reporter assay to determine whether TOP3B poisons and other compounds have antiviral activities (14). This assay is based on expression of a luciferase reporter inserted into MHV viral genome. We infected mouse 17CL-1 cells with 0.02 multiplicity of infection (MOI) MHV-Luc and then treated the infected cells with various compounds or dimethyl sulfoxide (DMSO, a negative solvent control). We subsequently detected luciferase activities in cell lysates at 20 h post infection (hpi). The host cells infected with 0.02 MOI MHV-Luc for 20 h attached to the bottom of the cultured wells and showed normal cell morphology and density, suggesting that most of the cells survived in this infection condition.

We first tested the representative compounds from the two families of TOP3B poisons: thiacyanine dye NSC96932 and bisacridine NSC690634 (Fig. 1*B*) in MHV inhibition, using remdesivir as a positive control (3, 15). Remdesivir at 0.1 μM concentration shows about 5-fold inhibition of MHV replication (Fig. 1*C*). NSC96932 at 0.0625 to 0.25 μM concentrations shows 6- to 30-fold inhibition (Fig. 1*C*), suggesting that it has higher potency than remdesivir. NSC96932 does not inhibit 17Cl-1 cell proliferation at concentrations lower than 0.25 μM but shows mild and significant inhibition of cell proliferation at 0.5 μM (Fig. S1). Because the antiviral activity of NSC96932 was observed at concentrations lower than 0.25 μM, its antiviral activity should not be due to its toxicity to the host cells.

The other TOP3B poison NSC690634, a bisacridine, does not inhibit MHV-Luc at 0.125 μM (Fig. 1*D*). At higher concentrations (0.25 and 0.5 μM), NSC690634 displays strong MHV inhibition (5- to 50-fold). However, the infected 17CL-1 cells are detached from the plate at these concentrations, suggesting that this compound at high concentrations is toxic to the host cells, which may lead to inhibition of virus amplification (Fig. 1*D*). Based on these results, we conclude that NSC96932 is a potent MHV inhibitor. We did not pursue further studies of NSC690634 due to its high toxicity in host cells.

### The anti-MHV activity of NSC96932 is independent of TOP3B

We next tested whether the antiviral activity of NSC96932 depends on TOP3B by examining whether this compound can inhibit MHV in *TOP3B*-KO 17Cl-1 cell lines previously generated (12). If the TOP3B-DNA and/or TOP3B-RNA cleavage complexes induced by NSC96932 are necessary for MHV inhibition, we predicted that NSC96932 should not inhibit MHV replication in *TOP3B*-KO 17Cl-1 cells. However, our results show that the antiviral activity of NSC96932 judged by the MHV-Luc assay in two independent *Top3b*-KO 17Cl-1 cell lines is indistinguishable from that of WT cells, as the compound reduces the MHV-Luc activity about 30-fold in all three cell lines (Fig. 1*E*). Thus, the anti-MHV activity of NSC96932 does not require TOP3B and may depend on other targets as proposed in Figure 1*A*.

### Multiple thiacyanines have potent anti-MHV activities

We next investigated whether the potent antiviral activity of NSC96932 can be observed for other thiacyanine dyes. We screened three groups of compounds with the MHV-Luc assay, using a cutoff of IC_50_ = 0.5 μM in the initial screen, and IC_50_ = 0.1 μM in the secondary screen. Group 1 consists of the known and potential inhibitors/poisons of topoisomerases 1 and 2, which have structures unrelated to thiacyanines and were not predicted to have antiviral activity (Fig. S2). As expected, our screening showed that 26 of 27 compounds of this group (96%) showed no antiviral activity (IC_50_ > 0.5 μM), indicating that our screening method is specific and can exclude vast majority of the screened compounds. Only one of 27 compounds (4%) shows IC_50_ lower than 0.5 μM (Fig. S2, marked by arrow), but this compound shows little or no MHV inhibition at 0.1 μM or 0.2 μM (data not shown). We did not continue studying this compound because its antiviral activity (based on IC_50_ of ∼0.5 M) is at least 10-fold weaker than that of NSC96932 and other members of the thiacyanine dyes (see below).

Group 2 compounds were chosen because they retain one of the two benzothiazole moieties present in thiacyanines with connection to a different ring moiety by linkers with variable sizes and shapes (Fig. S3*A*). Our screening showed that two of the 9 compounds (22%) have IC_50_ lower than 0.5 μM. However, only one of the two (∼11%), NSC179220, shows IC_50_ lower than 0.1 μM (Fig. S3*B*), whereas 8 out of 9 (89%) do not. Because the main structural difference between group 2 and thiacyanine dyes is that the former has one benzothiazole moiety, whereas the latter has two, these results suggest that two benzothiazole moieties are likely needed for strong antiviral activity. The only exception, NSC179220, has a saturated ring moiety connected to the benzothiazole moiety through a linker, in contrast to other compounds of this group that have unsaturated rings (Fig. S3*A*). It remains to be determined whether this saturated ring structure is critical for antiviral activity.

**Figure 2:**
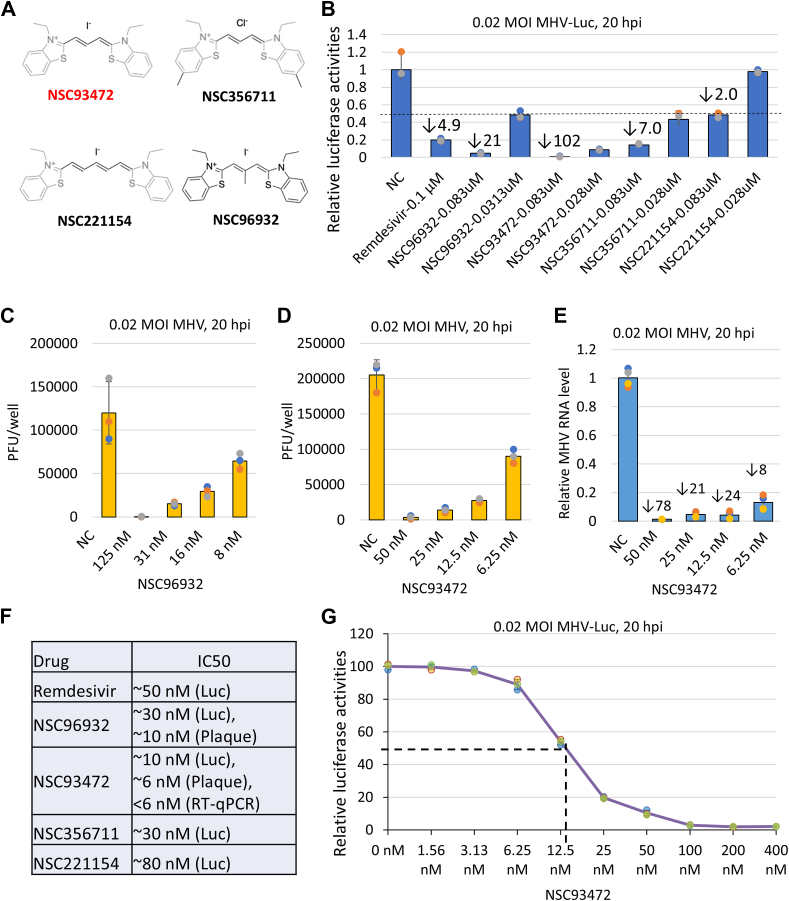
**NSC93472 has the strongest MHV inhibition effects among the screened thiacyanine dyes.***A*, structures of four thiacyanine dyes (NSC93472, NSC35711, NSC22153, and NSC96932). *B*, inhibition effects of MHV-Luc replication by four thiacyanine dyes. Remdesivir (Rem) was used as a positive control. Inhibition folds (>2) are indicated. *C*, inhibition effects of MHV replication by NSC96932 determined by plaque assay. For each data point, MHV titer from a well with infected cells (17Cl-1) treated with DMSO or a drug in a 96-well plate was calculated. *D*, inhibition effects of MHV replication by NSC93472 determined by plaque assay. *E*, inhibition effects of MHV replication by NSC93472 determined by RT-qPCR to detect MHV mRNA levels. *F*, estimated MHV inhibition IC_50_ of four thiacyanine dyes determined in MHV-Luc, plaque assay and RT-qPCR. *G*, curve of MHV-Luc inhibition by 0 to 400 nM NSC94372 to determine the precise IC_50_. DMSO, dimethyl sulfoxide; MHV, mouse hepatitis coronavirus; MHV-Luc, MHV-Luciferase; RT-qPCR, reverse transcription quantitative polymerase chain reaction.

Group 3 compounds were selected from the thiacyanine dye family and have similar structures as NSC96932, including 2 benzothiazole moieties connected by a carbon linker of various sizes and modifications. Our screening showed that all four thiacyanine dyes (three new ones plus NSC96932) exhibit robust antiviral activity, with their IC_50_ values all lower than 100 nM (Fig. 2, *A*, *B*, and *F*). Notably, three of the four thiacyanines (NSC93472, NSC356711, and NSC96932) have anti-MHV activity stronger than remdesivir, as their IC_50_ values are all lower than that of remdesivir (IC_50_∼50 nM in our assay), suggesting that the thiacyanine dye family may have potent antiviral activity in general. Together with the screen data of group 2 compounds, the structure of two linked benzothiazole moieties is likely essential for antiviral activity of the thiacyanines.

The linkers connecting the two benzothiazole moieties are different among the four thiacyanines with antiviral activity. The two (NSC93472 and NSC356711) with the same size of linker (three carbon-linker) as that in NSC96932 display the highest and the second highest anti-MHV activity, with IC_50_ values approximately 10 nM and 30 nM, respectively, which are comparable to or stronger than that of NSC96932 (IC_50_ ∼ 30 nM). In contrast, the dye NSC221154 with a longer linker (five carbon-linker) shows the lowest activity (IC_50_∼80 nM) (Fig. 2, *A*, *B*, and *F*), suggesting that the length of the linker could be crucial for antiviral activity of thiacyanines. Moreover, NSC93472 lacks the methyl group side chain present in the linker of NSC96932 and exhibits approximately 5-fold stronger antiviral activity, indicating that the side chain modification of the linker structure interferes with the antiviral activity.

We next investigated whether the antiviral activity of thiacyanine dyes revealed by the MHV-Luc assay could be confirmed by plaque assay. We examined two thiacyanine dyes, NSC93472 and NSC96932, which have the highest and the second highest antiviral activity from MHV-Luc assay, respectively. We found that in MHV-infected cells treated with 6.25 to 125 nM of either dye, MHV titers were reduced between 2- and 100-fold, when compared to DMSO (Fig. 2, *C* and *D*); and the IC_50_ values for both dyes are 10 nM or lower (Fig. 2, *C*, *D*, and *F*). These data agree with those from MHV-Luc assay (Fig. 2*B*), confirming that thiacyanine dyes possess potent antiviral activity. Between the two dyes, NSC93472 has greater activity than NSC96932, as its IC_50_ is lower than the latter (6 nM *versus* 10 nM) (Fig. 2, *C*, *D*, and *F*), which is also in agreement with the findings from the MHV-Luc assay. The representative plates with plaques, the original plaque counts and pfu calculations are shown in Fig. S4.

We further verified the antiviral activity of NSC93472 using reverse transcription quantitative polymerase chain reaction (RT-qPCR) analysis of MHV mRNA level. We found that in MHV-infected cells treated with 6.25 to 50 nM of NSC93472, the MHV mRNA level is reduced by 8- to 78-fold when compared to DMSO-treated cells, with an IC_50_ lower than 6 nM (Fig. 2, *E* and *F*). Together, the results from all three assays (MHV-Luc, plaque assay, and RT-qPCR) demonstrate that thiacyanine dyes have potent antiviral activity, with an IC_50_ as low as 6 nM (NSC93472).

To obtain the precise IC_50_ for NSC93472, we tested its MHV inhibition at a wide range of concentrations (0–400 nM) with 2-fold serial dilution by using MHV-Luc assay (Fig. 2*G*). We used 0 nM of the compound as the control to define 0% response level and 400 nM to define the 100% response level. These levels are defined properly, as no response was observed below 3 nM of the compound, and the maximal response was reached at 100 nM. The IC_50_ estimated from the curve is ∼13 nM. We also used Four Parameter Logistic Curve Calculator (AAT Bioquest) to calculate the IC_50_ and obtained IC50 of 13.1 nM. These values are close to the IC_50_ estimated by other methods (6–10 nM) (Fig. 2*F*), supporting the notion that NSC93472 is a potent inhibitor of MHV.

### NSC93472 shows anti-MHV activity in a mouse lung infection model

With NSC93472 showing the greatest inhibition potency in cell culture, we tested whether it shows similar bioactivity in an MHV-mouse model as previously described (12). We used two routes of administration for MHV and NSC93472 in C57BL/6J mice: intraperitoneal (IP) injection and intranasal (IN) inoculation, both of which have previously been used to infect mice with MHV (16).

**Figure 3:**
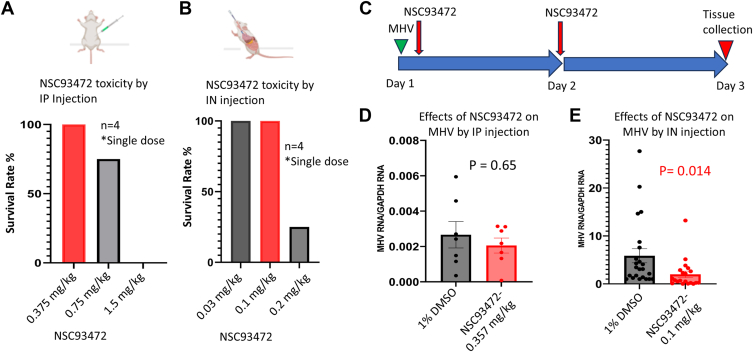
**NSC93472 shows significant MHV inhibition in mouse models by intranasal inoculation but not intraperitoneal injection.** Animals were anesthetized by intraperitoneal (IP) injection, and MHV and drugs were delivered by IP injection or IN inoculation. C57BL6 mice were used. *A*, schematics of IP injection and test of NSC93472 toxicity by IP injection. A single dose of NSC93472 was administrated, and four mice were tested in each group. The safe dose is 0.375 mg/kg. *B*, schematics of IN inoculation and test of NSC93472 toxicity by IN inoculation. A single dose of NSC93472 was administrated, and four mice were tested in each group. The safe dose is 0.1 mg/kg. *C*, schematics of time points for virus and drug delivery and tissue collection. *D*, NSC93472 at 0.375 mg/kg did not show significant MHV inhibition in mouse livers by IP injection. The mice were infected by 10^4^ pfu MHV through IP injection. MHV mRNA level in each mouse liver was detected by RT-qPCR and normalized by GAPDH mRNA. *p* value was calculated by *t* test with one-tailed distribution and two-sample equal variance. *E*, NSC93472 at 0.1 mg/kg showed significant MHV inhibition in mouse lungs by IN inoculation. The mice were infected by 10^5^ pfu MHV through IN injection. MHV mRNA level in each mouse lung was detected by RT-qPCR and normalized by GAPDH mRNA. *p* value was calculated by *t* test with one-tailed distribution and two-sample equal variance. IN, intraperitoneal; MHV, mouse hepatitis coronavirus; RT-qPCR, reverse transcription quantitative polymerase chain reaction.

We tested the maximum tolerated dose of NSC93472 by IP injection with three doses: 0.375 mg/kg, 0.75 mg/kg, and 1.5 mg/kg. Mice with 0.375 mg/kg dose had 100% survival rate, whereas the other two dosages led to lethality effects with 75% and 0% survival rates, respectively (Fig. 3*A*). We also tested the maximum tolerated dose of NSC93472 by IN inoculation with three doses: 0.03 mg/kg, 0.1 mg/kg, and 0.2 mg/kg. Mice inoculated with either 0.03 mg/kg or 0.1 mg/kg show 100% survival rate, whereas those with 0.2 mg/kg show 25% survival rate (Fig. 3*B*). With these toxicity data, we used the 0.375 mg/kg NSC93472 for IP injection and 0.1 mg/kg for IN inoculation, respectively.

In both IP and IN inoculation methods, MHV and first dose NSC93472 was sequentially delivered on day 1, NSC93472 was delivered again on day 2, and the livers or lungs were collected on day 3 for MHV detection by RT-qPCR (Fig. 3*C*). The results show that MHV levels in livers of the mice IP injected with either 1% DMSO (vehicle control) or NSC93472 do not show significant differences (Fig. 3*D*). However, we find that NSC93472 significantly inhibits MHV replication in the lungs of the mice treated by IN inoculation, reducing the average of MHV mRNA level by 3-fold compared to control mice treated with DMSO (*p* < 0.05) (Fig. 3*E*). These results demonstrate that NSC93472 inhibits MHV replication in the lung-infection model.

### NSC93472 binds RNA fragments derived from SARS-CoV-2 *in vitro*

**Figure 4:**
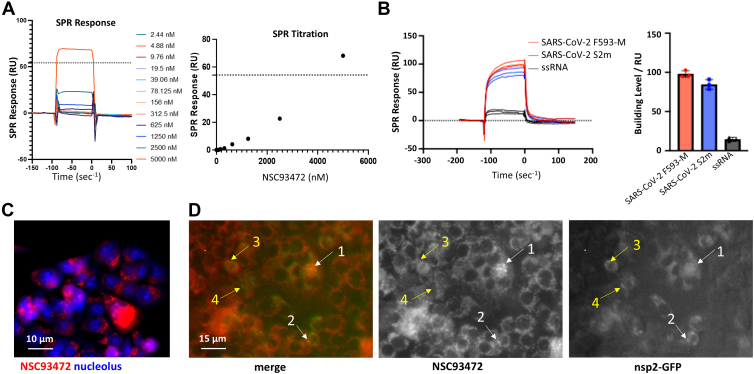
**NSC93472 interacts MHV RNA *in vitro* and *in vivo*.***A*, SPR response and titration curves for NSC93472 binding with SARS-CoV-2 F593-M RNA. NSC93472 shows binding greater than 1:1 at 5 μM. The *dashed line* indicates the theoretical max SPR response (R-max) for 1:1 binding event. *B*, SPR responses of NSC93472 binding with F593-M RNA and S2m RNA, and an ssRNA. *C*, NSC93472 localization in HEK293 cells. Cells were treated with 1 μM NSC93472 for 1 h and stained with nucleolar stain (ab139475). *D*, colocalization of NSC93472 and nsp2-GFP in 17Cl-1 cells infected by MHV-nsp2-GFP. 17Cl-1 cells were infected with 0.02 MOI MHV-nsp2-GFP (nsp2 is an MHV RdRp component) for 16 h, and then incubated with 0.2 μM NSC93472 for 8 h. The nsp2-GFP signals that are overlapped and nonoverlapped with NSC93472 in cells are indicated by *white* and *yellow arrows*, respectively. HEK293, human embryonic kidney 293 cells; MHV, mouse hepatitis coronavirus; MOI, multiplicity of infection; RdRp, RNA-dependent RNA polymerase; SPR, surface plasmon resonance.

Our findings that the antiviral activity of NSC93472 is independent of TOP3B prompted us to investigate whether thiacyanine dyes such as NSC93472 can directly bind viral RNA and/or RNA/protein complexes to inhibit viral replication. Previous studies have shown that some thiacyanine dyes can bind a variety of biomolecules, including DNA, RNA, and proteins (17, 18, 19). We specifically tested the RNA binding activity of NSC93472 to two RNA fragments derived from SARS-CoV-2 genome by surface plasmon resonance (SPR). The first fragment, named F593-M, is a modified form of a 78-nt RNA stem–loop structure from SARS-CoV-2 genome (F593) (20). We specifically replaced 57 nt from the 5′-end of the F593 with a new sequence to disrupt the stem–loop (see Experimental procedures), whereas leaving the 3′-21 nt sequence intact. RNA secondary structure prediction using RNAfold confirmed that the stability of stem–loops formed by F593-M is drastically reduced compared to F593 (ΔG = −1.6 *versus* −15.9 kcal/mol). The purpose of disrupting its stem–loop is to make it as a suitable RNA template in assay of the RdRp activity in the absence or presence of NSC93472 (see below). Thus, the SPR analysis using this fragment should reveal whether NSC93472 can bind the RNA template used in our RdRp assay. Our results showed that after dilution of NSC93472 at 12 different concentrations ranging from 5 μM to 2.5 nM, RNA-ligand binding is observed only at high concentrations (5 μM–312 nM) (Fig. 4*A*). The SPR response (resonance unit) exceeds theoretical maximum SPR response (R_max_ = 50.2 RU) for 1:1 binding event (dash lines, Fig. 4*A*) at 5 μM, indicating nonspecific RNA binding or more than one binding event per RNA for NSC93472.

The second RNA fragment tested for NSC93472 binding is the 47 nt stem–loop 2 motif (S2m) from SARS-CoV-2 (21). As a control, we included a 31-nt ssRNA consisting of a randomly selected sequence. RNAfold prediction revealed that this ssRNA cannot form a stable stem–loop (ΔG = 0 kCal/mol), whereas S2m can form a stable one (ΔG = −6.9 kCal/mol). SPR analysis revealed that NSC93472 displays binding response level to S2m similar to that of F593-M fragment (Fig. 4*B*). Moreover, the binding to both F593M and S2m are 4- to 5-fold stronger than that to the control ssRNA (Fig. 4*B*). This large difference in binding may not be simply due to the different lengths of the RNAs, as F593M (78 nt) and s2m (47 nt) are only 2.5- and 1.5-fold longer, respectively, than the ssRNA (31 nt). The difference may also not be due to presence or absence of a stem–loop, because F593M has a disrupted stem–loop, whereas S2m has a stable one, yet the former has slightly higher drug binding than the latter. One likely explanation of the difference is that NSC93472 may preferentially bind unknown sequence and/or structural motifs present in F593M and S2m but absent in the ssRNA.

We also compared the RNA-binding activity of NSC93472 with that of three other control compounds, using the same F593M RNA as the substrate. Among the control compounds, Neomycin and Thiazole Orange, as nonspecific RNA binders, were utilized as positive controls. PreQ1, as a natural and specific ligand for PreQ1 riboswitch, was used as a negative control. As expected, NSC93472, Neomycin and Thiazole Orange bind F593M at 5 μM, whereas PreQ1 shows little or no binding (Fig. S5). The data support the notion that NSC93472 may act as a nonspecific binder for RNA.

### NSC93472 partially colocalizes with MHV replication machinery in cytoplasm

To study whether NSC93472 can bind MHV *in vivo*, we investigated whether the dye is present in the cytoplasm and colocalizes with the viruses in the infected cells. NSC93472 showed red color under the fluorescent light, and its signals were largely excluded from the nucleolus, marked by nucleolar staining (ab139475) (Fig. 4*C*), suggesting that NSC93472 is mainly localized in the cytoplasm. The largely cytoplasmic localization of NSC93472 implies that the dye may mainly interact with RNA or RNA–protein complexes, which are more abundant in cytoplasm than nucleus. Alternatively, the dye may not emit red fluorescence signals in nucleus. Our findings that NSC93472 can bind SARS-CoV-2 derived structured RNAs *in vitro* and partially colocalize with viral replication machinery in cells (see below) favor the first possibility.

We next tested whether NSC93472 colocalizes with MHV replication machinery by co-staining the dye with a nsp2 and GFP fusion protein (nsp2-GFP), which is a nonessential component of MHV RdRp (14). The images of MHV-nsp2-GFP–infected and NSC93472-treated cells showed that their signals overlap in some cells (Fig. 4*D*). To further characterize the colocalization of NSC93472 and nsp2-GFP, we obtained the intensity reads of both channels in individual nsp2-GFP–positive areas and generated a dot plot to calculate Pearson's correlation coefficient value (R) (Fig. S6). The R value from all samples is 0.06, indicating a very weak or no correlation between these two signals. However, in the selected areas with NSC93472 intensities greater than 90 (Fig. S6, boxed area), the two signals displayed moderate positive correlation (R = 0.41). These data suggest that NSC93472 may colocalize with and possibly interact with the viral replication machinery at some but not all stages of the MHV lifecycle.

We also tested whether NSC93472 affects mRNA translation using a rabbit reticulocyte lysate *in vitro* translation system. By comparing with DMSO negative control, NSC93472 at 0.1 μM and 0.5 μM did not show translation inhibition for luciferase mRNA or MHV-Luc mRNA (Fig. S7). As a control, the translation of these mRNAs is strongly inhibited by cycloheximide at 0.5 μM (Fig. S7). Based on RNA-binding assay by SPR, NSC93472 displayed significant RNA-binding activity at 0.5 μM but not 0.1 μM (Fig. 4*A*), whereas the dye did not inhibit translation at either concentration, suggesting that RNA binding of NSC93472 does not exert significant inhibition of mRNA translation. These results do not support the possibility that NSC93472 inhibits MHV replication by suppressing viral RNA translation.

### NSC93472 inhibits RdRp of SARS-CoV-2 and reverse transcriptase of MMLV *in vitro*

**Figure 5:**
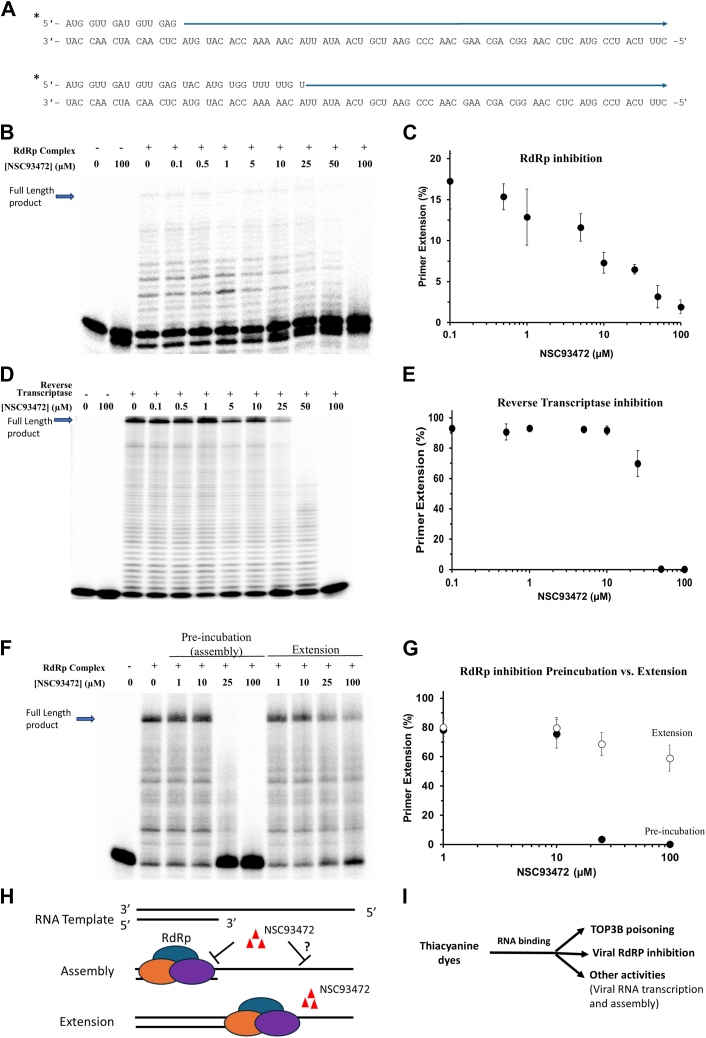
**NSC93472 inhibits RdRp and reverse transcriptase activities.***A*, F593-M RNA primer-template substrates used for Nsp12/7/8 primer extension assays. The *asterisk* at the 5′ end of the top strand indicates the position of the ^32^P-radiolabel. The *arrow* depicts RNA synthesis extension. *B*, Nsp12 (700 nM), Nsp7 (7200 nM), Nsp8 (7200 nM), and the F593-M RNA substrate (80 nM) were incubated with NSC93472 or DMSO for 120 min at 30 °C. Representative denaturing gel of the NSC93472 titration is shown. *C*, quantitative assessment of RdRp primer extension activity (all RNA extension products) affected by different concentrations (μM) of NCS93472. Data represent an average of three independent experiments with SD indicated by error bars. *D*, reverse transcriptase enzyme and the F593-M substrate annealed to the 15-nt DNA primer (80 nM) was incubated with NSC93472 or DMSO for 15 min at 37 °C. Representative denaturing gel of the primer extension with a NSC93472 titration. *E*, quantitative assessment of reverse transcriptase activity (all DNA extension products) affected by different concentrations (μM) of NCS93472. Data represent an average of three independent experiments with SD indicated by error bars. *F*, Nsp12 (400 nM), Nsp7 (7200 nM), Nsp8 (7200 nM), and the F593-M RNA substrate with a 31-nt RNA primer (80 nM) were incubated with NSC93472 or DMSO during a 15 min preincubation step or extension initiation step with addition of rNTPs to initiate the reaction for 60 min at 30 °C. Representative denaturing gel of the NSC93472 titrations under both conditions is shown. *G*, quantitative assessment of RdRp primer extension activity on the longer RNA primer-template substrate (all RNA extension products) affected by different concentrations (μM) of NCS93472 when added during the preincubation (*closed circle*) or extension initiation (*open circles*). Data represent an average of three independent experiments with SD indicated by error bars. *H*, the model of NSC93472’s effects on assembly and extension of RdRp on an RNA template. *I*, schematics of activities of thiacynine dyes as TOP3B poison, RNA replicase inhibitor, and others. These activities may depend on their RNA-binding abilities. DMSO, dimethyl sulfoxide; MHV, mouse hepatitis coronavirus; MHV-Luc, MHV-Luciferase; RdRp, RNA-dependent RNA polymerase.

Our findings that NSC93472 binds RNA *in vitro* and partially colocalizes with RdRP of MHV in cells imply that it may directly inhibit the viral replication machinery. We examined this hypothesis by testing the compound’s ability to inhibit RdRP activity using an *in vitro* primer extension assay. Briefly, we purified the recombinant core RdRp components and reconstructed its RNA synthesis activity according to published protocol (Fig. S8) (22). We performed a serial dilution of the compound and added the indicated concentration of compound to a reaction mixture containing RdRP, the RNA template with the annealed 15 nt RNA primer (Fig. 5*A*), and ribonucleoside triphosphates (rNTPs). We examined all extension products at NSC93472 concentrations between 0 to 100 μM (Fig. 5, *B* and *C*). Notably, at 50 and 100 μM, NSC93472 partially or nearly completely eliminated the extension products, respectively, indicating that it inhibits SARS-CoV-2 RdRP, with an IC_50_ of less than 50 μM.

We also studied the effects of NSC93472 for inhibition of reverse transcriptase (RT) of Moloney murine leukemia virus (MMLV), which is an essential enzyme in retroviruses and functions in reverse transcribing ssRNA into dsDNA. NSC93472 showed no inhibition at concentrations below 10 μM, with no measurable reduction in the various primer extension products observed (Fig. 5, *D* and *E*). However, NSC93472 displayed partial inhibition of the primer extension products at 25 μM, strong inhibition at 50 μM, and complete inhibition at 100 μM (Fig. 5, *D* and *E*), indicating that it can inhibit RT with an IC_50_ of approximately 50 μM. These data resemble those from RdRP (Fig. 5, *B* and *C*), suggesting that NSC93472 can directly inhibit replication machinery from different RNA viruses.

### NSC93472 interferes with assembly of the elongation-competent complex of RdRp

Our findings that NSC93472 preferentially binds the F593M RNA used as a template in the primer extension assay over a control ssRNA (Fig. 4*B*) imply that the compound may bind the RNA substrate to inhibit assembly of the elongation-competent complex formed by RdRp binding to the same RNA. To examine this hypothesis, we divided the primer-extension assay into two stages: a preincubation stage without addition of rNTPs to allow assembly of the RdRp onto RNA; and an extension stage when all four rNTPs are added to allow RNA synthesis to occur. We also doubled the length of the primer from 15 nt to 31 nt to increase the length of the duplex RNA region before the first nucleotide of incorporation (Fig. 5*A*). The reason is that RdRp of SARS-CoV-2 contacts about 2 turns of duplex RNA before the first nucleotide of incorporation (22). The 15-nt primer can only form a 1.4 turns of duplex RNA, which may be too short for full contacts with RdRp to form a stable elongation-competent complex. The 31-nt primer can form more than 2 turns of duplex region and should be able to have full contacts with RdRp to form a more stable elongation-competent complex.

We made the following observations using the new primer and the modified conditions. First, the RdRp proteins were preincubated with the RNA primer-template substrate for increasing periods of times (5 min–4 h), and primer extension was subsequently initiated with the addition of rNTPs (Fig. S9). We observed that the new 31-nt primer has substantially increased the amounts of full-length extension products than the 15-nt primer (compare Figs. 5*F* and S9 with 5B), consistent with our rationale above that the longer duplex region may allow assembly of a more stable elongation-competent complex. Second, we found that there was no improvement in primer extension with longer preincubation times, suggesting that the RdRp proteins assemble efficiently onto the RNA primer-template substrate during the shortest preincubation period conducted (5 min) to catalyze the extension (Fig. S9). Third, we observed stronger dose-dependent reduction of the levels of extension products by NSC93472 when the drug was added at the onset of the preincubation stage, compared to the condition in which the drug was added at the onset of primer extension stage (Fig. 5, *F* and *G*). For example, the relative levels of the extension products were reduced from 80% to about 2% and 0%, respectively, when 25 and 100 mM of NSC93472 was added at the onset of the preincubation stage, respectively. In contrast, the relative levels of the extension products were reduced from about 80% to only 70% and 60%, respectively, when the same concentrations of the drug were added at the onset of extension period. These data suggest that NSC93472 may interfere with RdRp assembly onto the RNA substrate to form the elongation-competent complex. However, our data do not exclude the possibility that the drug may alter the RNA template structure during the preincubation time and thus interfere with the extension step (Fig. 5*H*).

To determine if NSC93472 exerts inhibition on initial primer extension products similar to its effects on the full-length products, we preincubated RdRp with NSC93472 and the RNA template, but added only ATP to the reaction which should only be incorporated at the first two positions after the primer. We found that the RdRp complex was able to extend the primer further than the first two nucleotides complementary to the first two uridines in the template (Fig. S10), suggesting compromised fidelity in the absence of the Nsp10-14 proofreading complex. This extension, however, is short in length and failed to produce any full-length products (compare Figs. S9 with S10). Under these conditions, NSC93472 (25 or 100 μM) inhibited RdRp primer extension activity, with its pattern (strong inhibition at 25 μM) similar to what was observed in the presence of all four rNTPs (Fig. S10 *versus* Fig. 5*F*, left). The data are in agreement with the conclusion from the preincubation experiment (Fig. 5, *F* and *G*) that NSC93472 may interfere with RdRp assembly on the RNA primer-template substrate, resulting in inhibition of initial nucleotide incorporation similar to its effects on the full extension products.

To address the relative potency of NSC93472, we compared its effects on RdRp synthesis to that of PSI-7409, which is a nucleoside analog and a nonobligate chain terminator. PSI-7409 is also the active form for sofosbuvir, an FDA-approved drug to treat Hepatitis C virus, an RNA virus of Flaviviridae family (23). Sofosbuvir has been used in small clinical trials to treat Coronavirus disease 2019 patients and was found to reduce mortality rate and improve the clinical outcome (24). We preincubated either PSI-7409 or NSC93472 with RdRp and the RNA primer-template and assessed primer extension activity upon addition of rNTPs. We found that PSI-7409 reduced the relative levels of the primer extension products by about 2.5-fold, from about 75% to 30% at 25 μM concentrations (Fig. S11, *A* and *B*). This reduction is about 4-fold weaker than that of NSC93472, which reduced the levels of the extension products by about 10-fold at 25 μM concentration. These findings reinforce the notion that NSC93472 is a potent inhibitor of RdRp-mediated viral RNA synthesis.

## Discussion

Previous studies have shown that knockout of *TOP3B* inhibits replication of several positive-sense RNA viruses (11). Although our recent studies demonstrated that knockout of *TOP3B* does not suppress replication of MHV, which is also a positive-sense RNA virus (12), it is still plausible that induction of TOP3B–viral RNA cleavage complexes by poisoning drugs can inhibit replication of MHV and other RNA viruses. To test this hypothesis, we used our established anti-MHV drug screen model to test the activities of two families of TOP3B poisons identified recently (3). We found that one family of TOP3B poisons, the thiacyanine dyes, shows potent MHV inhibition activities. Specifically, three thiacyanine dyes, NSC96932, NSC93472, and NSC356711, not only stand out from the screen but also have greater anti-MHV activities than remdesivir. Moreover, one of the dyes, NSC93472, can inhibit MHV replication in a mouse model. Furthermore, from our initial screening of compounds that inhibit human SARS-CoV-2 and Zika virus, we found that the thiacyanine dye NSC96932 strongly inhibits their replication at 1 μM concentration (Fig. S12). Thus, our data suggest that thiacyanine dyes could be candidates to be developed as antiviral drugs for multiple RNA viruses.

Our data suggest that the antiviral effect of thiacyanine dyes is mediated through targets other than TOP3B (Fig. 1*E*). Several lines of evidence support a model that these dyes may bind viral RNA to interfere with the assembly of an elongation-competent complex formed by RdRp binding to the same viral RNA (Fig. 5*H*). First, our *in vitro* binding assays show that NSC93472 preferentially binds two RNA fragments derived from SARS-CoV-2 genome over a random ssRNA (Fig. 4, *A* and *B*). Second, the fluorescence imaging shows that the localization of NSC93472 is mainly in cytoplasm and partially overlaps with that of MHV RdRp, suggesting that the dye may interact with viral replication machinery in cells (Fig. 4, *C* and *D*). Third, our primer-extension assays demonstrate that the thiacyanine dyes can inhibit the activities of RdRp of SARS-CoV-2 and RT of MMLV (Fig. 5, *A*–*G*). Fourth, adding NSC93472 prior to the assembly of the RdRp–RNA template complex results in stronger inhibition of RdRp activity than adding it immediately before the extension step (Fig. 5, *F* and *G*). Fifth, NSC93472 can strongly inhibit RdRp extension reaction for the initial products (when only the rNTP for the first 2 nucleotides is added to the reaction) (Fig. S10), and this effect is similar to that for extension of the full products (when all four rNTPs are present) (Fig. 5*B*). Together, our data favor a hypothesis that the antiviral activity of thiacyanine dyes is mediated by its binding to viral RNA, and this binding interferes with the binding of RdRp to viral RNA to form an elongation-competent complex (Fig. 5*H*). Because this mechanism does not involve formation of TOP3B–RNA cleavage complex, the antiviral activity and poisoning of TOP3B should be two independent actions of NSC93472 and other thiacyanine dyes (Fig. 5*I*).

We noted that NSC93472 is more potent in inhibiting MHV replication in cell line–based assays (luciferase and plaque assays) than RdRP-based biochemical assay (IC_50_ in nM *versus* μM range). One explanation for this big difference in potency is that the thiacyanine dyes may act through mechanisms other than inhibiting RdRP. For example, NSC93462 may bind structured viral RNAs and inhibit other steps of virus life cycle, such as viral RNA transcription and viral particle assembly (Fig. 5*I*). Alternatively, this difference could be due to the inability of our *in vitro* RdRP assay to fully recapitulate the dynamic cellular environment where viral replication occurs.

Although our results demonstrate that thiacyanine dyes inhibit MHV at low concentrations without significant cell toxicity, higher concentrations of thiacyanine dyes do show toxicity in cultured host cells and animals. For example, NSC93472 can cause toxicity in mice at concentrations between 0.2 and 1 mg/kg, which is about 50-fold more toxic than remdesivir which can be used safely in mice at 50 mg/kg. Future studies are needed to address this toxicity issue by either modifying the drugs to reduce side effects, or by using better delivery methods. It should be mentioned that one of thiacyanine dyes, NSC221154, has been used therapeutically in human and dogs to treat broad-spectrum parasite worm infections (25). In addition, the dye can significantly increase survival in a rat model of glioblastoma when delivered using brain-penetrating nanoparticles (26). NSC221154 has weaker but still potent anti-MHV activity as assessed by the MHV-Luc assay (IC_50_ = 80 nM) compared to the three other thiacyanines (IC_50_ = 10–30 nM). Thus, it may be possible to find a suitable thiacyanine with a balance of antiviral activity and tolerable toxicity, or use a safer delivery method, to treat patients infected with RNA viruses.

## Experimental procedures

### Cell culture and virus infection

Mouse 17Cl-1 cells and L2 cells were kindly provided by Dr Paul Masters (Wadsworth Center) and Dr Susan Weiss (University of Pennsylvania), respectively. The cell lines were maintained in Dulbecco’s modified Eagle’s medium (DMEM) (Gibco) with 10% fetal bovine serum (FBS) at 37 °C with 5% CO2. MHV-A59 virus was kindly provided by Dr Susan Weiss. MHV-nsp2-GFP and MHV-Luc were kindly provided by Dr Mark Denison (Vanderbilt University Medical Center). L2 cells were mainly used in plaque assay, and 17Cl-1 cells were used in most of other MHV infection experiments. For MHV infection, 17Cl-1 cells were grown in 24-well or 96-well plates to reach confluency, washed with PBS once, infected with MHV in DMEM for 1 h, replaced with fresh medium without and with a compound, and incubated for indicated time in each experiment.

### Plaque assay

To prepare the viral samples for plaque assay, the 96-well plates with cultured cell samples in 100 μl medium were frozen and thawed twice, the samples were centrifuged at 4000*g* for 10 min, and the supernatant was used for plaque assay. Plaque assays were carried out with plates covered with confluent L2 cells to determine MHV titer (27). Briefly, 10 μl supernatant of each sample was first diluted by 990 μl DMEM, and two subsequent 1:10 dilutions in DMEM were made. Two hundred microliters diluted samples with 1:10 serial dilutions were used to infect 6-well plate covered with L2 cells. The wells were covered with soft agar, and the wells with distinct clones were used for counting. The value of pfu/well for each sample was calculated according to the number of plagues and dilution.

### RNA extraction and RT-qPCR

Total RNA was extracted from MHV-infected 17Cl-1 cells, mouse lungs, and livers with TRIzol (Invitrogen). complementary DNA (cDNA) was synthesized from 1 μg RNA using Taqman Reverse Transcription Reagents (Applied Biosystems). After 1:3 dilution, 1 μl cDNA was used as a template for a 20 μl qPCR with SYBR Green PCR Master Mix (Applied Biosystems). MHV mRNA levels are normalized by GAPDH mRNA levels. qPCR primers are:

MHV-ORF1a-F: 5′-GAGGTCAGAGGAGGATGGGT-3′

MHV-ORF1a-R: 5′-TGGCAGACTGAACACAGCAT-3′

GAPDH-F: 5′-AACTTTGGCATTGTGGAAGG-3′

GAPDH-R: 5′-GGATGCAGGGATGATGTTCT-3′

### Chemical screens with MHV-Luc

All the compounds used in this study were obtained from Developmental Therapeutics Program of National Cancer Institute. Remdesivir was purchased from MedChemExpress LLC (Cat#: NY-104077). The compounds at 1 μM or lower concentrations were dissolved in 0.1% DMSO. 17CL-1 cells were infected by 0.02 MOI MHV-Luc in a 96-well plate and incubated in medium with compounds and 0.1% DMSO as negative control for 20 h. The samples were washed with PBS once, lysed with 50 μl 1XPLB lysis buffer (Promega)/well, shook for 10 min at room temperature, frozen in −80 °C for 20 min, and then shook for 15 min at room temperature. After adding 100 μl luciferase reagent (Promega) into each well and pipetting, 100 μl mixed solution was moved into a 96-well luciferase plate, and the luciferase signals were detected in a 96-well luciferase reader (Promega).

#### Detection of NSC93472 and nsp2-GFP subcellular localization

Human embryonic kidney HEK293 cells were treated with 1 μM NSC93472 for 1 h, washed out with regular media, and stained with nucleolar stain (ab139475). To detect the colocalization of NSC93472 and MHV-nsp2-GFP, 17Cl-1 cells were infected with 0.02 MOI MHV-nsp2-GFP for 16 h, incubated with 0.2 μM NSC93472 for 8 h, and washed once with PBS. Pictures were taken immediately under a Zeiss Axiovert 200 fluorescence microscope. To quantify the fluorescent signals, individual nsp2-GFP positive areas were selected, and the intensity values of green (nsp2-GFP) and red (NSC93472) intensities were calculated by color histogram tool of ImageJ (https://imagej.net/ij/). Pearson correlation coefficient rate is calculated by an online calculator (https://www.socscistatistics.com/tests/pearson/default2.aspx).

#### Drug toxicity in mice

C57BL/6j eight-week-old female mice were used for drug toxicity tests. Two routes of inoculations were used: IN inoculation and IP injection. Four groups of four mice were used to test NSC93472 toxicity by IN inoculation. Group one received 1% DMSO as a control, while groups 2 to 4 received 0.03 mg/kg, 0.1 mg/kg, and 0.2 mg/kg NSC93472, respectively. A single dose of 30 μl DMSO or drug was equally dropped into both nostrils of the mouse nose. Mice were observed for 72 h post drug inoculation to test for survival rates. Four groups of four mice were used to test NSC93472 toxicity by IP injection. Group 1 received 1% DMSO as a control, while groups 2 to 4 received 0.375 mg/kg, 0.75 mg/kg, and 1.5 mg/kg NSC93472, respectively. A single dose of 150 μl drug or DMSO was injected using 26 ½ gauge needle and 1 ml syringe. Mice were observed for 72 h post drug injection for survival rates.

#### MHV infection of mice

C57BL/6j eight-week-old female mice were used for MHV inoculation and drug effects tests. Mice were anesthetized with 20 μl/g ketamine/xylazine cocktail (2.5 mg/ml ketamine + 0.25 mg/ml xylazine) in 1× PBS. For IN inoculation route, two groups of 23 mice were infected with 10^5^ pfu of MHV diluted in 30 μl DMEM. The liquid with viruses was equally dropped using a mechanical pipette into both nostrils of the mouse nose. Mice were observed until the liquid was completely inhaled. Thirty minutes post MHV inoculation, group 1 was given 1% DMSO as a negative control, while group 2 was treated with 0.1 mg/kg of NSC93472 (30 μl) *via* IN inoculation. Mice were observed after anesthesia until they regain normal activity. Twenty-four hours post MHV inoculation, drug was administrated following the same route mentioned above. Lungs were harvested 72 hpi. For IP injection route, two groups of seven mice were infected with 10^5^ pfu of MHV diluted in DMEM. The virus was injected intraperitoneally. Thirty minutes post infection, group 1 was given 1% DMSO as a negative control, while group 2 was treated with 0.375 mg/kg of NSC93472 (1% of the mouse body weight which was around 150 μl) *via* IP injection. Twenty-four hours post infection, a second dose of the drug was administrated. Livers were harvested 72 hpi. All animal procedures were approved by the NIA Animal Care and Use Committee and followed the NIH animal guidelines.

### Surface plasmon resonance

SPR binding assays were performed with a BIAcore 3000 (GE Healthcare) instrument. A CM5 SPR biochip was docked into the instrument and primed with running buffer (25 mM Tris pH 7.5, 100 mM KCl, 0.2 mM MgCl_2_, 0.005% Tween-20, 5% DMSO). The chip surface was generated in three steps. First, flow cells (F_C_1-F_C_4) were activated with an injection of an aqueous solution of EDC/NHS (0.4 M/0.1 M) at a flow rate of 5 μl/min for 15 min. Then, a solution of streptavidin (0.2 mg/ml in 10 mM sodium acetate buffer, pH 4.5) was injected for 30 min. After the immobilization reached ∼6000 RU, the surface was quenched by flowing 1 M ethanolamine (pH 8.5) for 10 min, followed by 10 mM NaOH for 2 min to remove streptavidin that was not covalently linked to the chip surface.

For RNA immobilization, a 5 μM solution RNA was annealed in annealing buffer (25 mM Tris pH 7.5, 100 mM KCl, 0.2 mM MgCl_2_, 0.005% Tween-20) at 95 °C for 3 min and flash-cooled on ice. After the RNA was incubated on ice for 1 h, the RNA solution was diluted to 1 μM and injected into individual flow cell for 30 min at a flow rate of 5 μl/min to reach an RNA immobilization level of ∼2700 RU. In this study, biotinylated F593-M RNA template strand was used as a target (F_C_2), and two more RNAs (SARS-CoV-2 S2m, F_C_3, and a random ssRNA, F_C_4) were used as controls. The RNA sequences are shown in an M&M section below. The flow rate was then increased to 25 μl/min, and small molecule solutions were not injected until the baseline was stable.

For ligand-RNA binding, analysis was performed at a flow rate of 25 μl/min. NSC93472 was diluted in DMSO to 200 μM and then further diluted in an annealing buffer, resulting in a final concentration of 5% DMSO. NSC93472 solution was serially diluted (1:1) in running buffer from 5 μM to 2.5 nM. Fifty microliters of each dilution was injected in all flow cells for 120 s association. Dissociation was set at 200 s, and if needed, a 50 μl injection of 1 M KCl was used to regenerate the baseline between samples. In parallel, three control compounds (Neomycin, Thiazole orange, and PreQ_1_) were also tested at 5 μM. The binding signal was obtained by reference subtraction (F_C_1) using BIAevaluation 4.0 (https://www.pubcompare.ai/product/JXWyNZcByx5TsEHUI--g/), and the final SPR response (RU) for concentration was plotted in GraphPad Prism 10 (https://www.graphpad.com/updates/prism-1000-release-notes).

#### Recombinant proteins used for *in vitro* primer extension assays

Plasmids pET-His-SCov2-nsp7 and pET-His-SCov2-nsp8 for Nsp7 and Nsp8 protein expression were a gift from Patrick Cramer (Addgene plasmid 154757 and 154758, respectively) (22). The plasmid pFastBac-HTB-nsp12-FLAG for Nsp12 expression was transformed into DH10Bac competent cells (Thermo Fisher Scientific) to create baculovirus DNA with Nsp12 coding sequence. MMLV RT was purchased from New England BioLabsInc. The detailed methods to purify Nsp7, Nsp8, and Nsp12 proteins are as following.

Plasmid pET-His-SCov2-nsp7 or pET-His-SCov2-nsp8 was transformed into *Escherichia. coli* strain BL21-CodonPlus (DE3)-RIL (Agilent). Bacteria were grown in LB (1 L culture) supplemented with ampicillin (100 μg/ml) and chloramphenicol (34 μg/ml) to OD_600_ = 0.6. Protein expression was induced with 0.5 mM IPTG for 18 h at 18 °C. Cells were harvested by centrifugation (6000*g*, 10 min, 4 °C) and resuspended in 20 ml of buffer A (50 mM Hepes-NaOH, pH 7.5, 500 mM NaCl, 10% glycerol, 5 mM β-mercaptoethanol [BME]) supplemented with 25 mM imidazole and Complete EDTA-free protease inhibitors (Roche). Bacteria were lysed using Branson Digital Sonifier for 5 min in ice bath (30 s On/30 s Off duty cycle). The lysate was clarified by centrifugation (40,000*g*, 10 min, 4 °C) and filtered through 0.45 μm polyvinylidene fluoride membrane. Chromatography steps were performed using AKTA pure system (Cytiva). Clarified lysate was loaded onto 5 ml HisTrap HP column equilibrated with buffer A + 25 mM imidazole. The column was washed stepwise with 10 column volumes (CVs) of buffer A + 50 mM imidazole and buffer A + 100 mM imidazole. Next, the protein was eluted with 10 CV of buffer A + 250 mM imidazole, eluted fractions were concentrated and exchanged into TEV protease buffer (50 mM Hepes-NaOH, pH 7.5, 10% glycerol, 5 mM BME) using Amicon Ultra-4 5K MWCO concentrator. The final protein concentration was measured using Pierce 660 nm protein assay (Thermo Fisher Scientific), TEV protease (New England BioLabs) was added according to manufacturer’s instructions and incubated overnight at 4 °C. The protease reaction was loaded onto 5 ml HisTrap HP column equilibrated with buffer A + 25 mM imidazole and flow-through containing cleaved protein was collected and concentrated to the final volume of 1 ml. The concentrate was loaded onto HiLoad 16/60 Superdex 200 prep grade column (Cytiva) equilibrated with buffer B (20 mM Hepes-NaOH, pH 7.5, 150 mM NaCl, 5% glycerol, 5 mM Tris(2-carboxyethyl)phosphine hydrochloride) and developed with 1.5 CV at 1 ml/min flow rate. Fractions containing purified protein were pooled, concentrated with Amicon Ultra-4 5K MWCO concentrator, aliquoted, flash-frozen in liquid nitrogen, and stored at −80 °C. Purity of the recombinant Nsp7 and Nsp8 proteins was determined to be at least 90% homogeneous (Fig. S5*A*).

pFastBac-HTB-nsp12-FLAG was transfected into 8 × 10^5^ *Spodoptera frugiperda* (Sf9) insect cells with Cellfectin II (Thermo Fisher Scientific) in 6-well dish. The insect cells were grown in Grace’s Insect Cell Medium supplemented with 10% FBS and 46 U/ml pen/46 μg/ml strep, and virus production proceeded in the 6-well dish until most of the cells were lysed to generate low-titer baculovirus. A small amount of this low titer baculovirus was isolated and used to infect 2 × 10^7^ Sf9 cells in a flask to produce high-titer baculovirus. A total of 0.5 ml of high-titer baculovirus from the previous step was added to 10 T175 flasks containing 2 × 10^7^ Hi5 insect cells (Thermo Fisher Scientific) each, grown in Express Five SFM Medium supplemented with 16 mM L-glutamine and 46 U/ml pen/46 μg/ml strep, 40 h after infection, the infected Hi5 insect cells were centrifuged at 500*g* for 8 min in 250-ml conical-bottom centrifuge tubes (Nalgene), and the media were removed. Cell pellets were washed with 35 ml 1x PBS and transferred to 50-ml conical tubes (Falcon), followed by centrifugation at 500*g* for 5 min. The wash was repeated with 25 ml 1x PBS containing protease inhibitor without EDTA (Roche) and centrifuged at 500*g* for 5 min. Cell pellets were stored at −80 °C until used for protein affinity purification. Cell pellets containing His-Nsp12-FLAG protein were purified using the same buffers, columns, and methods as our His-Nsp13-FLAG purification (28). The only exception is that due to Nsp12 being larger than Nsp13 we used a molecular weight cut-off filter of 50 kD for all dialysis and concentration steps in the purification. The purified Nsp12 was stored in the same storage buffer as Nsp13 (100 mM Tris–HCl, pH 8.0, 100 mM NaCl, 10% glycerol, 5 mM BME) and stored at −80 °C. Protein concentration was estimated, and samples from each step of the purification and the final protein sample were analyzed by electrophoresis using a Bis-Tris 4 to 12% gradient polyacrylamide gel followed by Coomassie staining. Purity of the recombinant Nsp12 protein was determined to be at least 90% homogeneous (Fig. S5*B*).

#### SARS-CoV-2 replication–transcription complex and RT primer extension assays

For SARS-CoV-2 RdRp primer extension assays, Nsp12 (400–700 nM), Nsp7 (7200 nM), and Nsp8 (7200 nM) were incubated with the indicated cyanine dye, PSI-7409 tetrasodium (Medchem Express), or the solvent carrier DMSO (concentration indicated in figure legend) and the 78-nt F593-M template RNA substrate annealed to either a 15-nt or 31-nt RNA primer (80 nM). Oligonucleotides used for Primer Extension Assays are shown below. The 10 μl reaction mixtures contained 10 mM Tris–HCl (pH 7.5), 10 mM KCl, 2 mM MgCl_2_, 1 mM DTT, and 0.25 mM rNTPs. Nsp7 and Nsp8 were added to reaction mixtures on ice, followed by the addition of Nsp12. The enzymes were incubated for 2 min at room temperature to prevent precipitation of the subsequently added NSC93472. After adding NSC93472, the reactions were incubated for 5 to 15 min at 30 °C. Primer extension reactions were initiated by the addition of rNTPs and with incubation at 30 °C for 60 to 120 min. Six microliters of proteinase K was added to the reaction mixtures for 15 min at room temperature. Reaction mixtures were quenched with the addition of 16 μl FLB stop buffer (80% formamide, 0.1% xylene cyanol, 0.1% bromophenol blue, 5% 10× TBE). Reaction products were denatured for 5 min at 95 °C and 2 μl loaded and resolved on 12% polyacrylamide (7 M urea) denaturing gels electrophoresed at 40 W for 1 h at room temperature. Gels were exposed on a Phosphorimager screen and scanned with a Typhoon FLA 9500 Imager. ImageQuantTL software (https://info.cytivalifesciences.com/image-analysis-software.html) was utilized for quantification of gel images. For RT (0.15625 U/ul) primer extension assays, reactions included the F593-M RNA template annealed to the equivalent 15-nt DNA primer. The enzyme and 10× reaction buffer originated from New England BioLabs_Inc_ M-MuLV Reverse Transcriptase kit. Addition of the RT initiated the same 2-min incubation at room temperature, followed by addition of the NSC93472 for a 5-min incubation at 37 °C. After addition of the dNTPs (0.25 mM each), the reactions were incubated for 15 min at 37 °C. Reactions were quenched at 65 °C for 20 min, and 10 μl FLB stop buffer was added. Gel electrophoresis resolution and analysis of the RT reaction mixture products followed the same procedures used for products of the SARS-CoV-2 RdRp reaction mixtures.

#### Oligonucleotides used to prepare substrates for SPR and primer extension assays

SARS-CoV-2 F593-M RNA Template Strand: 5′-CUUUCAUCCGUACUCCAAGGCAGCA AGC AACCCGAAUCGUCAAUAUUACAAAAACCACAUGUACUCAACAUCAACCAU-3′

15-nt RNA Primer: 5′-AUGGUUGAUGUUGAG-3′

15-nt DNA Primer: 5′-ATGGTTGATGTTGAG-3′

31-nt RNA Primer: 5′-AUGGUUGAUGUUGAGUACAUGUGGUUUUUGU-3′

SARS-CoV-2 S2m: 5′- CAUUUUCACCGAGGCCACGCGGAGUACGAUCGAGUGUAC AGUGAACA-3′

ssRNA: 5′-UGUCCCCACACCCCUGUCCCCACACCCCUG U-3′

#### Cell culture, viral stocks, compound treatment, and plaque assays for Zika and SARS-CoV-2 viruses

Vero E6 cells (American Type Tissue Collection, C1008) and Vero CCL-81 were maintained in DMEMcontaining 10% FBS, 100 U/ml penicillin, 100 ug/ml streptomycin, and nonessential amino acids at 37 °C in a humidified incubator containing 5% CO_2_. Zika Dakar 41525 (ZIKV-Dakar), virus strain was kindly provided by Scott Weaver (UTMB Arbovirus Reference Collection) and propagated on Vero cells. The SARS-CoV-2 virus was propagated from a previously reported cDNA clone of USA-WA1/2020 SARS-CoV-2 in a BSL-3 laboratory (29). Briefly, the full-length cDNA was assembled *via in vitro* ligation and used as a template for T7 *in vitro* transcription to obtain the full-length viral RNA for electroporation into Vero E6 cells. The original P0 virus was harvested from the electroporated cells and propagated on Vero E6 cells to produce the P1 virus. The P1 virus was confirmed by sequencing and the viral titer was determined by plaque assay on Vero E6 cells, and the P1 virus was used for infection in this experiment.

Vero E6 cells (2 *×* 10^4^) were seeded onto collagen-coated Thermo-Nunc 96-well, black wall, optical bottom microwell plates with DMEM supplemented with 2% FBS, 1% P/S, and 1% nonessential amino acids. Twenty-four hours later cells were pretreated with either compound NSC96932 or vehicle DMSO and positive control NITD-008. Approximately 2 h after addition of compounds cells were infected with Zika Dakar at a MOI of 0.2 or SARS-COV-2 at a MOI of 0.01 for 1 to 2 h. Virus was then removed, cells washed in 1× PBS, and compounds were added again. Thirty-six to forty-eight hpi supernatants were collected for plaque assay to determine viral titers, and cells were analyzed for percent infection and cell viability by immunofluorescence.

ZIKV stocks and supernatants were analyzed by plaque assay in Vero cells. Vero cells (1 *×* 10^5^) were seeded into 24-well plates and cultured at 37 °C, 5% CO_2_ overnight. Virus was serially diluted in DMEM with 2% FBS and incubated with cells at 37 °C, 5% CO_2_ for 1 h. Virus was then removed, cells washed with PBS, and DMEM overlay media containing 2% FBS, 0.025 M Hepes, and 0.8% carboxymethylcellulose was added. At the fourth day post infection, the overlay was removed; cells were fixed in 4% formaldehyde, rinsed in dH_2_0, and stained with 1% crystal violet. Plaques were counted on a light box. SARS-COV-2 stocks and supernatants were analyzed by plaque assay in Vero E6 cells. Vero E6 cells (1.2 × 10^6^) were seeded into 6-well plates and cultured at 37 °C, 5% CO_2_ for 16 h. Virus was serially diluted in DMEM with 2% FBS and incubated with cells at 37 °C with 5% CO_2_ for 1 h. After the incubation, overlay medium was added to the infected cells. The overlay medium contained DMEM with 2% FBS and 1% sea-plaque agarose. After a 2-day incubation, the second overlay containing 0.33% neutral red was added and plaques were counted on a light box.

## Data availability

All data will be shared upon requests to the corresponding author.

## Supporting information

This article contains supporting information.

## Conflict of interest

The authors declare that they have no conflicts of interest with the contents of this article.
