# Supplementary material for: Discovery of thiacyanine dyes as a new class of potent coronavirus inhibitors that suppress viral RNA synthesis
Source: J Biol Chem. 2025 Aug 5;301(9):110547. doi: 10.1016/j.jbc.2025.110547 (PMC12423399; doi:10.1016/j.jbc.2025.110547)
Supplement: Supplementary Figures [file mmc1.pdf]

Figure S1. Cell toxicity of NSC96932 and NSC93472

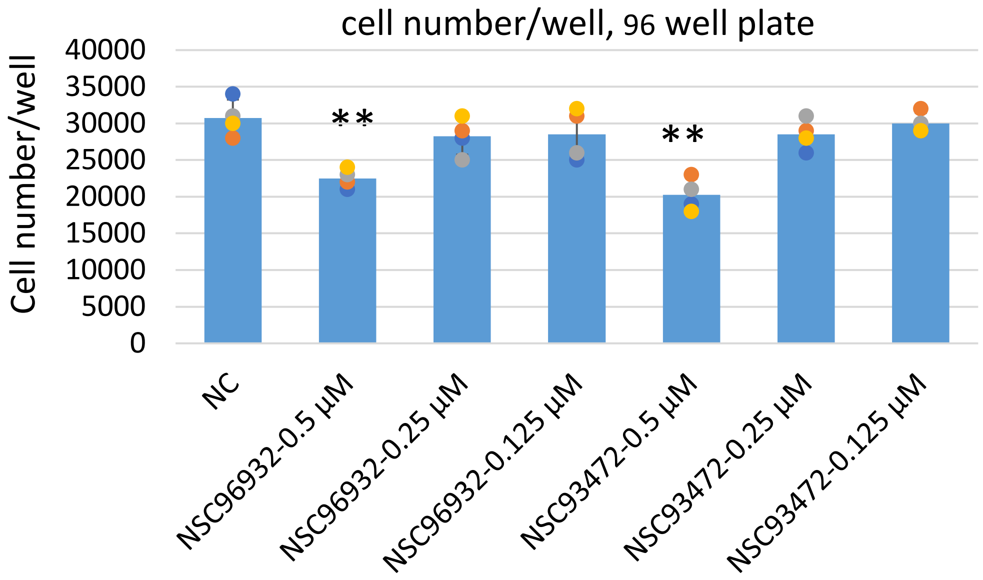

Cell number of 17Cl-1 from each well treated with compounds or 0.1% DMSO as NC after 24h incubation was calculated to determine cell toxicity. Significant decreases by t-test (P<0.01) are indicated by **.

Figure S2. Screen of MHV inhibition effects by compounds with TOP1 or TOP2 inhibition activities

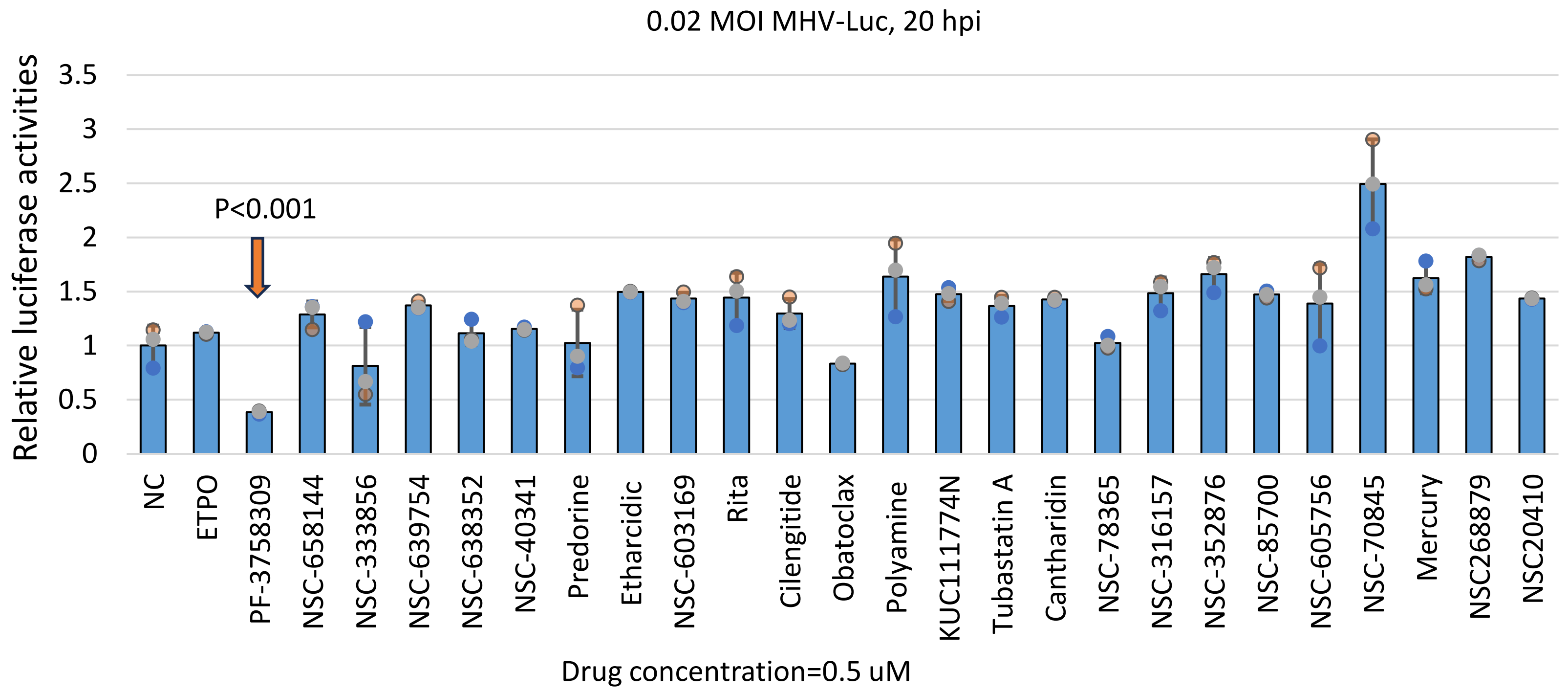

Inhibition effects of MHV-Luc replication by different compounds at 0.5 µM concentration. The only compound with > 2-fold inhibition is indicated by an arrow. P-value from t-test indicates significant decrease.

Figure S3. Screen of MHV inhibition effects by compounds with benzothiazole group

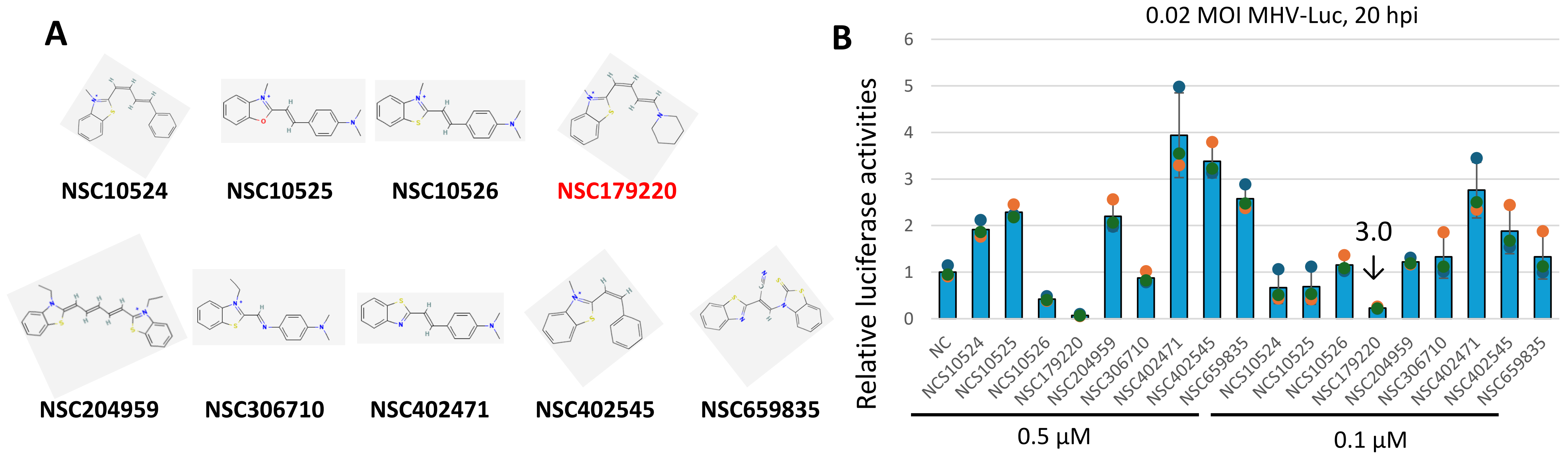

A) Structures of compounds with a Benzothiazole group. NSC179220 with anti-MHV activities is highlighted with red color. B) Inhibition effects of MHV-Luc replication by different compounds at 0.1 and 0.5 µM concentrations. Inhibition fold by NSC179220 at 0.1 µM is indicated.

Figure S4. Plague assay to detect MHV virus titers

A

10 μl out of 100 μl/well → 1 ml DMEM → 1:10 → 1:10

200 μl 200 μl 200 μl

B

Dilution: 1/50 1/500 1/5000

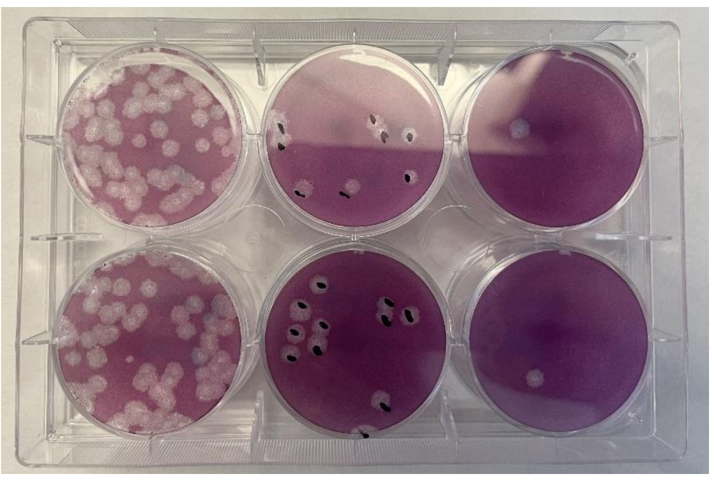

C

NSC96932	plague number	dilution	pfu/well
NC-1	18	5000	90000
NC-2	22	5000	110000
NC-3	32	5000	160000
125 nM-1	4	50	200
125 nM-2	6	50	300
125 nM-3	8	50	400
31 nM-1	26	500	13000
31 nM-2	34	500	17000
31 nM-3	30	500	15000
16 nM-1	70	500	35000
16 nM-2	60	500	30000
16 nM-3	46	500	23000
8 nM-1	13	5000	65000
8 nM-2	11	5000	55000
8 nM-3	15	5000	75000

NSC93472	plague number	dilution	pfu/well
NC-1	43	5000	215000
NC-2	36	5000	180000
NC-3	44	5000	220000
50 nM-1	12	500	6000
50 nM-2	30	50	1500
50 nM-3	70	50	3500
25 nM-1	35	500	17500
25 nM-2	21	500	10500
25 nM-3	28	500	14000
12.5 nM-1	56	500	28000
12.5 nM-2	49	500	24500
12.5 nM-3	60	500	30000
6.25 nM-1	20	5000	100000
6.25 nM-2	16	5000	80000
6.25 nM-3	18	5000	90000

A) samples for plague assay are diluted by 1/10 to three concentrations. B) A representative plate for plaque assay. C) The original data of calculation of pfu/well based on plaque numbers.

Figure S5. NSC93472 and two other non-specific RNA binders bind F596 RNA

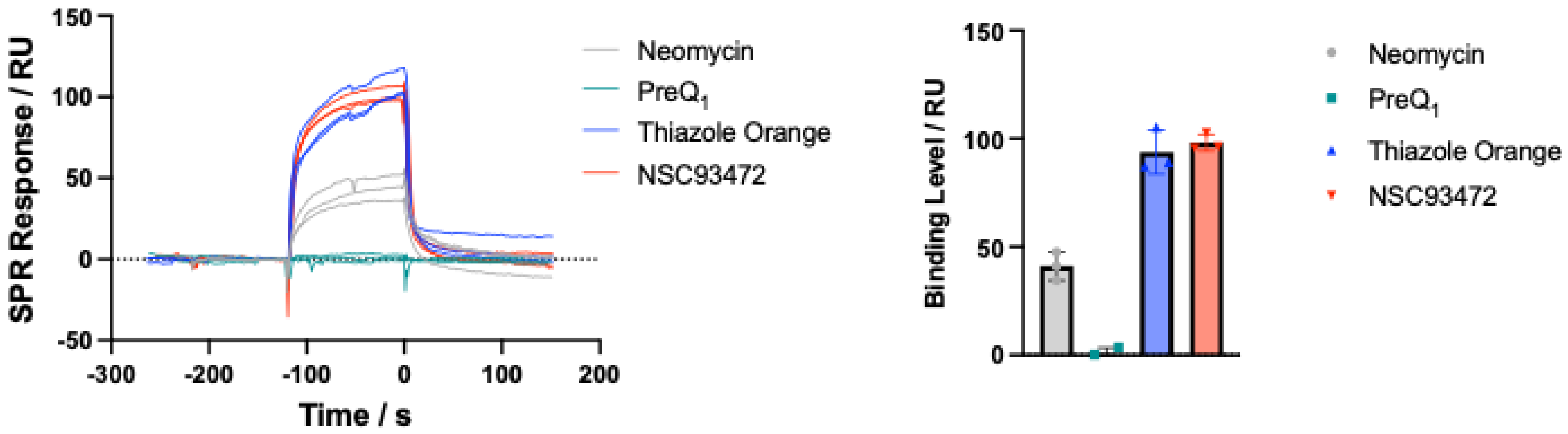

Aside from the key compound (NSC93472), three RNA-binding molecules were used as controls, including Neomycin (non-specific RNA binder), Thiazole Orange (non-specific RNA binder), and PreQ1 (only binds PreQ1 riboswitch aptamer). All the compounds were injected at 5 μM (n=3).

Figure S6. Correlation of NSC93472 and nsp2-GFP fluorescent signals

A

B

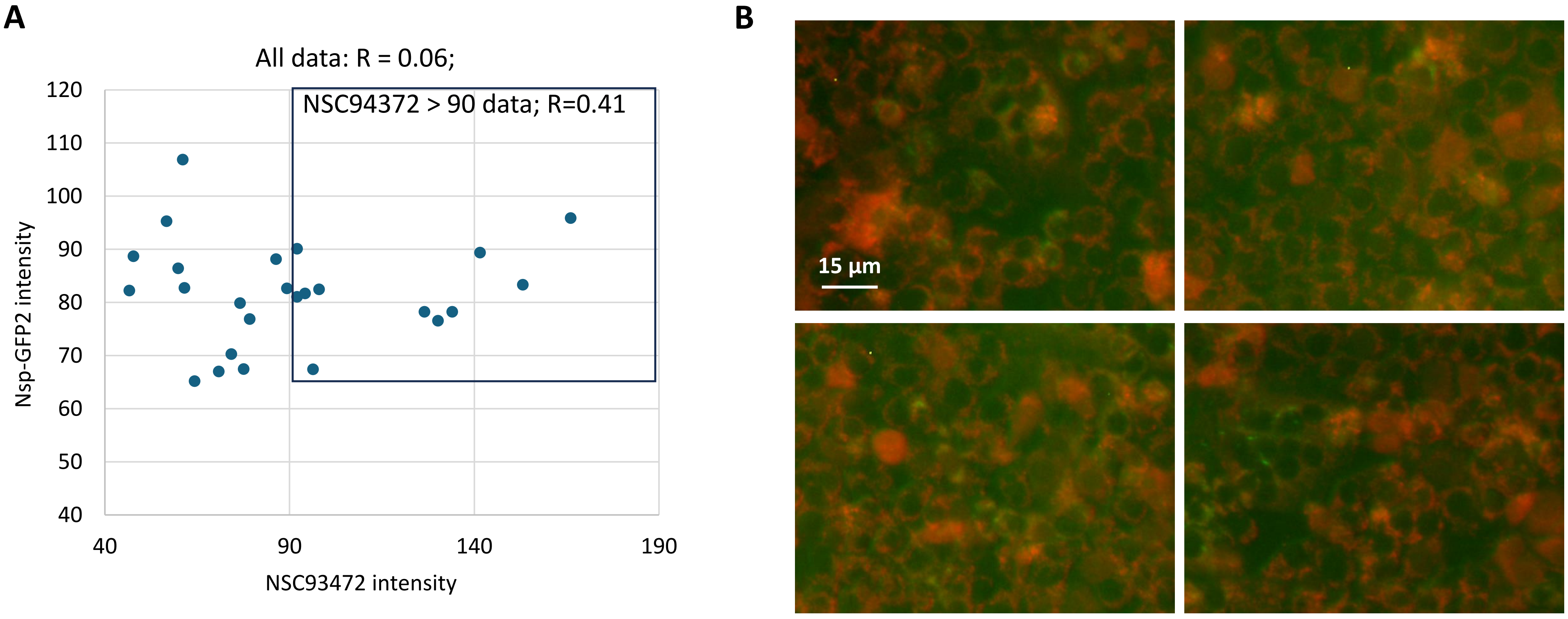

A) Dot plot of NSC93472 and nsp2-GFP intensities and the Pearson Correlation Coefficient value (R). The box marked area are data with NSC93472 signals> 90, in which the two signals display modest positive correlation (R=0.41). B) Representative images used for analysis.

Figure S7. NSC93472 does not affect Luciferase or MHV-Luc mRNA translation in a vitro assay

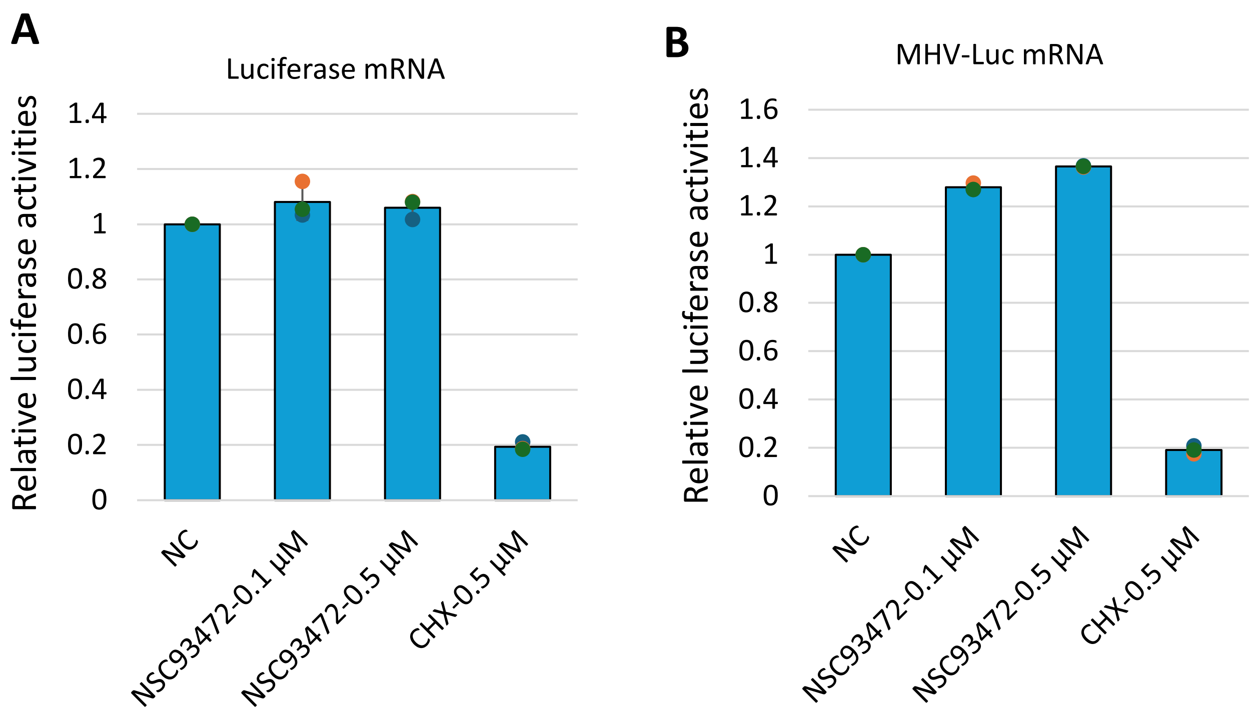

The translation efficiencies are determined by the luciferase activities in the rabbit reticulocyte lysate samples with Luciferase mRNA (A) or MHV-Luc mRNA (B). The mRNA, compound and rabbit reticulocyte lysate were mixed and incubated at 30°C for 1.5h before testing Luciferase activities. DMSO (0.1%) only and cycloheximide (CHX) are used as negative (NC) and positive controls, respectively.

Figure S8. Purification of Nsp7, Nsp8 and Nsp12

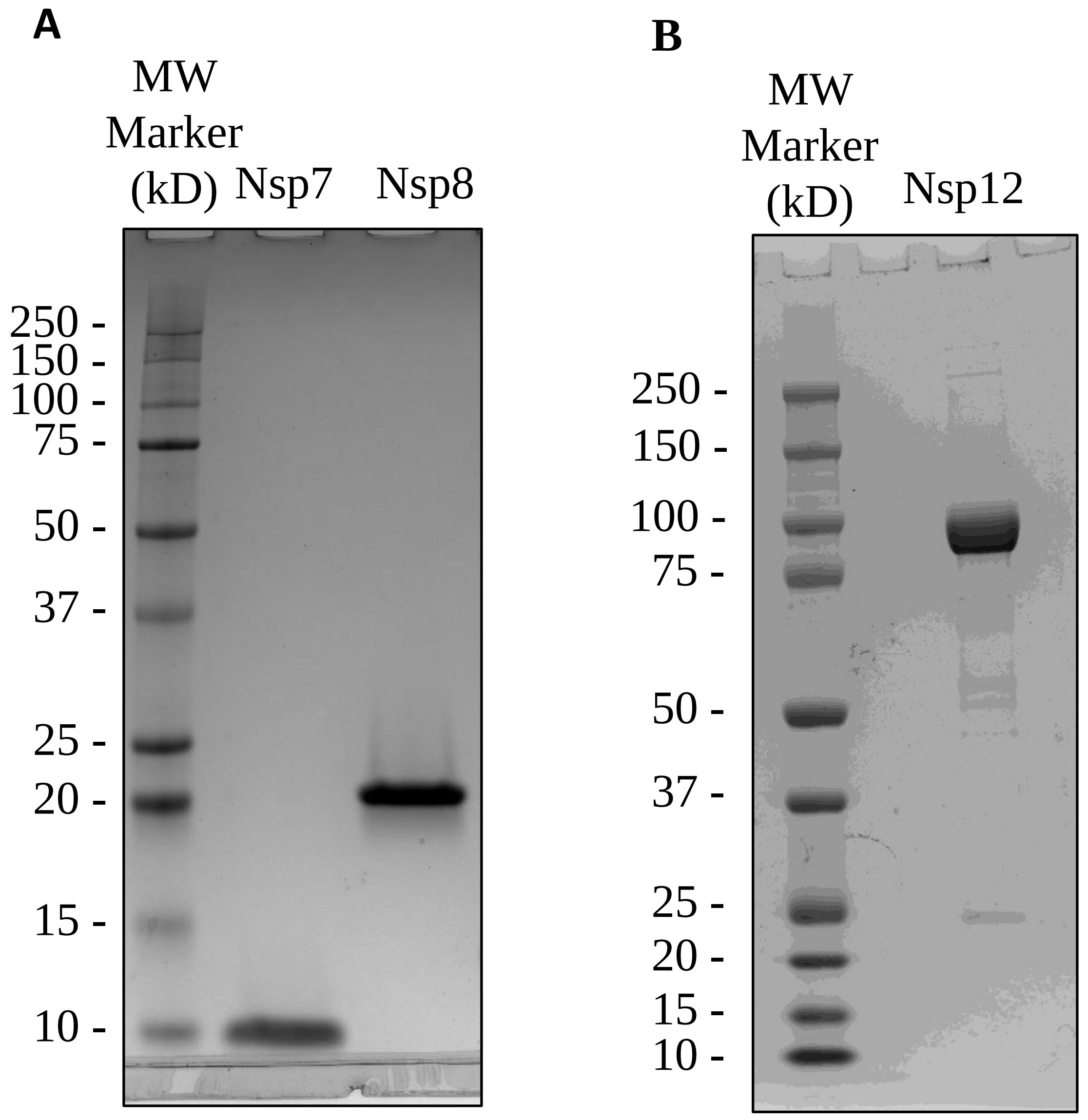

Nsp7 (1 µg), Nsp8 (1 µg) (A) and Nsp12 (3 µg) (B) after final dialysis (see Experimental procedures), resolved by SDS-PAGE and detected by Coomassie staining.

Figure S9. Assessment of pre-incubation times on RdRp synthesis

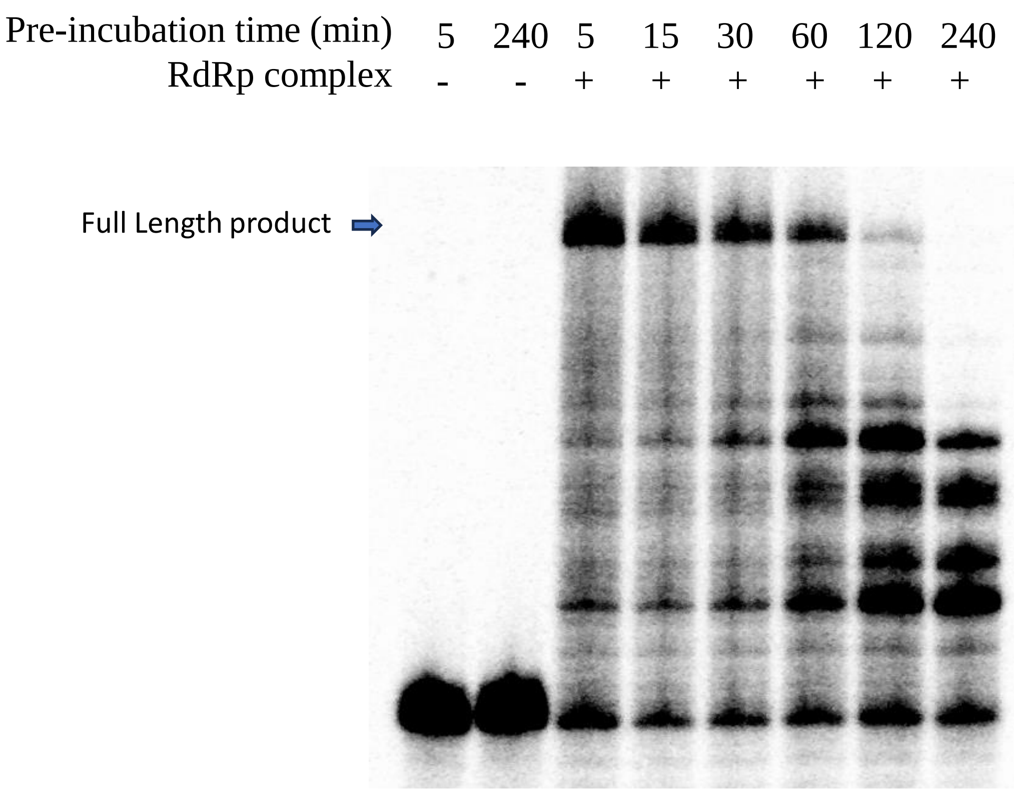

Nsp12 (400 nM), Nsp7 (7200 nM), Nsp8 (7200 nM) and the F593-M RNA substrate with a 31 nt RNA primer (80 nM) were pre-incubated incubated with increasing amounts of time prior to the addition of rNTPs to initiate the reaction for 60 min at 30°C. Representative denaturing gel of the NSC93472 titration is shown.

Figure S10. NSC93472 inhibits RdRp extension during initial nucleotide incorporation

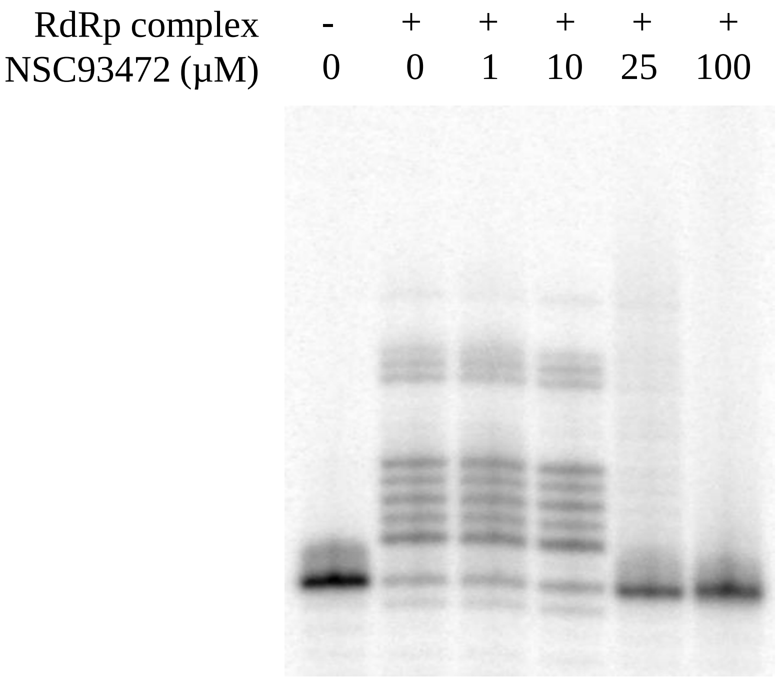

Nsp12 (400 nM), Nsp7 (7200 nM), Nsp8 (7200 nM) and the F593-M RNA substrate with a 31 nt RNA primer (80 nM) were incubated with increasing concentrations of NSC93472 (1, 10, 25 and 100 µM) during a 15 min preincubation step followed by the addition of only ATP to initiate the reaction for 60 min at 30°C. Representative denaturing gel of the NSC93472 titration is shown.

Figure S11. NSC93472 more potently inhibits RdRp synthesis compared to the non-obligated chain terminator PSI-7409

A

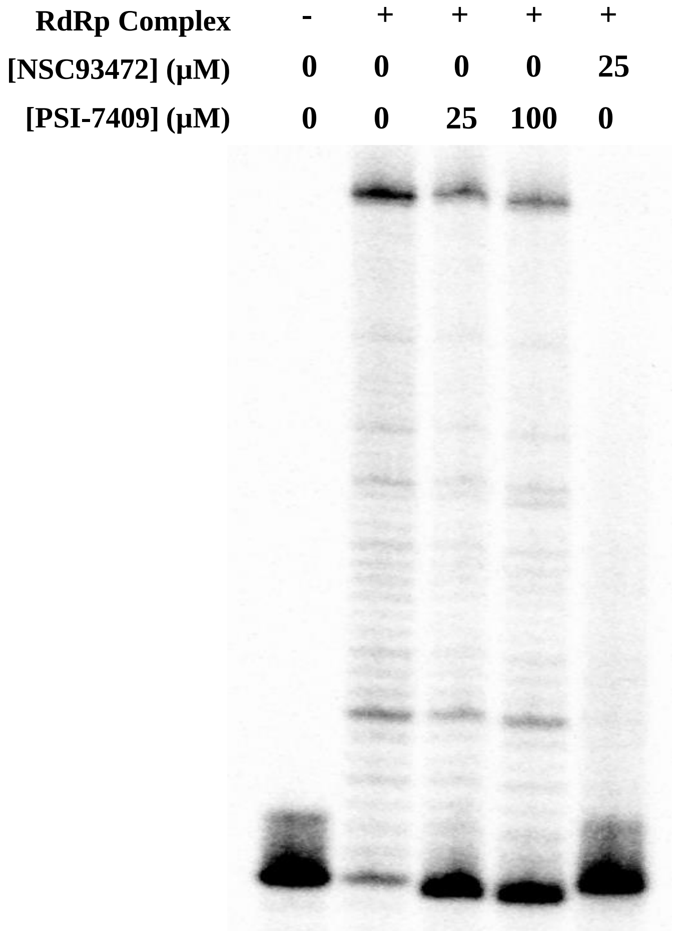

B

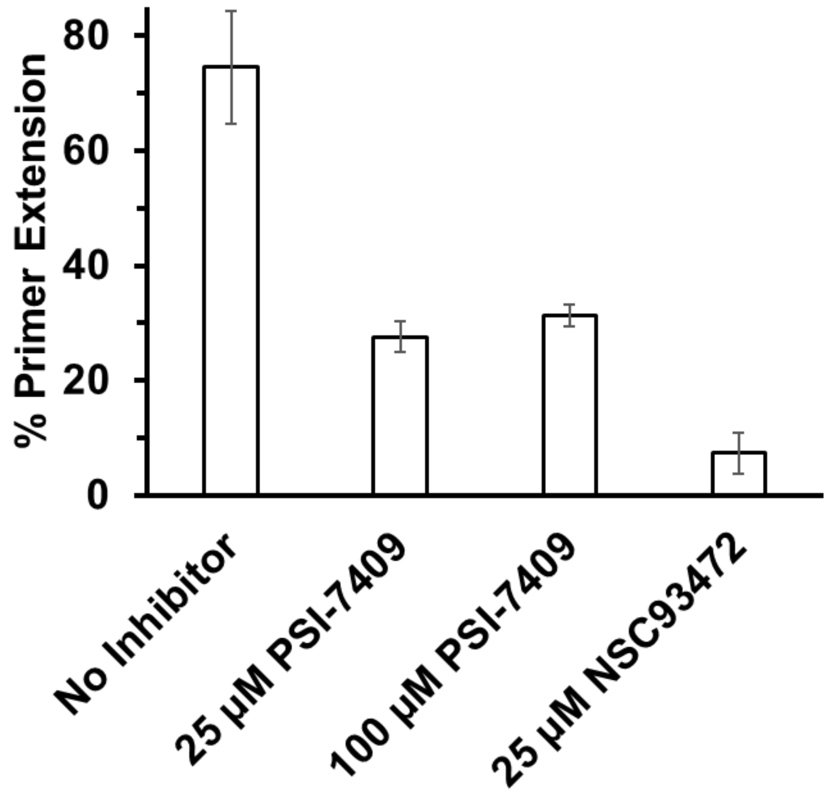

A) Nsp12 (400 nM), Nsp7 (7200 nM), Nsp8 (7200 nM) and the F593-M RNA substrate with a 31 nt RNA primer (80 nM) were incubated with NSC93472, PSI-7409 or DMSO during a 15 min preincubation step followed by the addition of rNTPs to initiate the reaction for 60 min at 30°C. Representative denaturing gel of PSI-7409 at two concentrations and a single NSC93472 concentration is shown. B) Quantitative assessment of RdRp primer extension activity on the longer RNA primer-template substrate (all RNA extension products) affected by different concentrations (µM) of PSI-7409 and NSC93472 when added during the preincubation. Data represent an average of three independent experiments with SD indicated by error bars.

Figure S12. Inhibition of SARS-CoV-2 and ZIKV by thiacyanine dye NSC96932

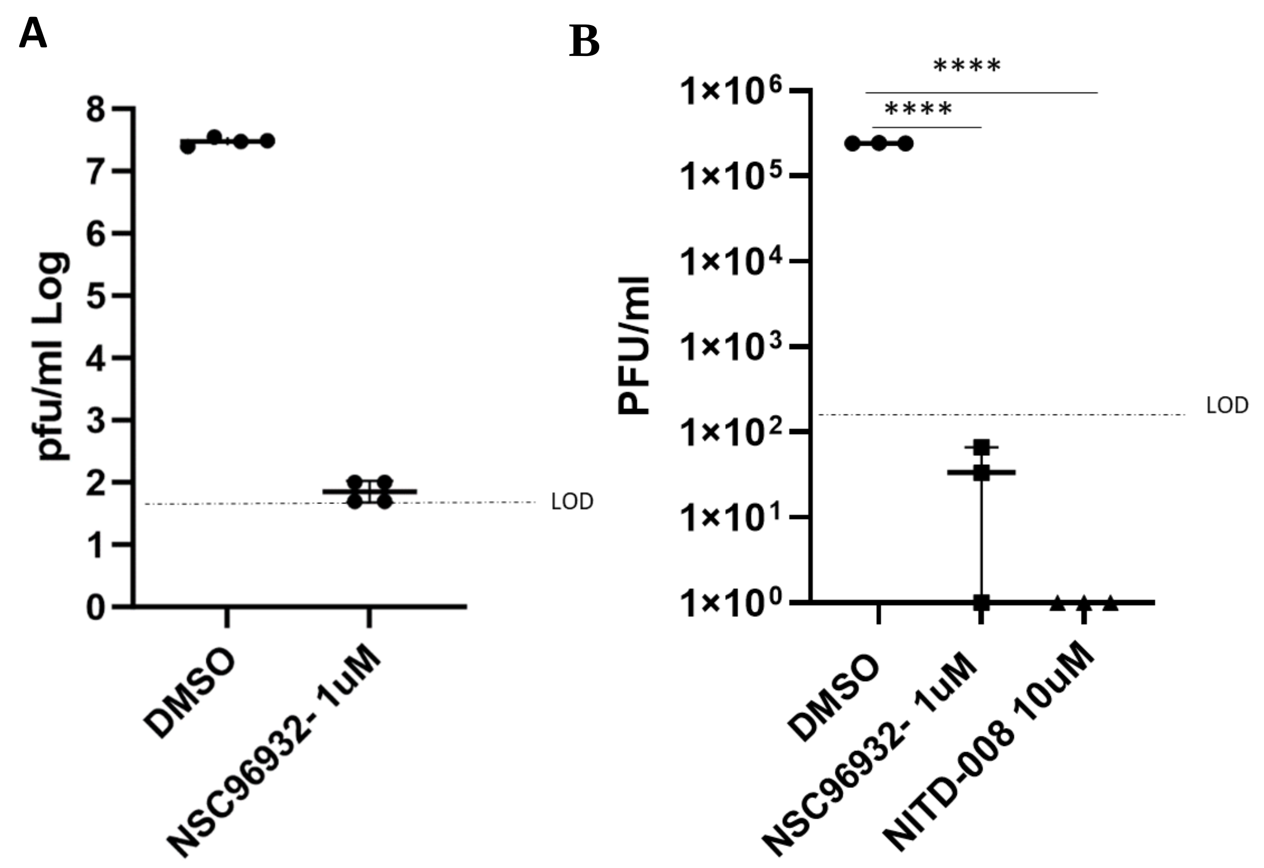

A) Effects of Thiacyanine dye NSC96932 on SARS-COV-2 virus infection (MOI 0.01) in Vero E6 cells. The graph displays viral titers at 36 hpi (pfu/ml) in supernatants of Vero E6 cells treated with 1uM of compound NSC96932 and vehicle (DMSO) B) Effects of Thiacyanine dye NSC96932 on Zika Dakar virus infection (MOI 0.2) in Vero E6 cells. The graph displays viral titers at 48 hpi (pfu/ml) in supernatants of Vero E6 cells treated with 1uM of compound NSC96932, vehicle (DMSO) and 10uM of positive control (NITD-008) from the same experiment. Data are represented as mean +/- standard deviation. Asterisks represent level of statistical significance *** $p<0.001$ and **** $p < 0.0001$. An Ordinary One-way ANOVA was performed with multiple comparisons in each experimental condition. Vero E6 cells treated with NSC96932 and infected with Zika Dakar were also assayed for percent infection and cell viability by immunofluorescence. Viral infection was reduced from 30% in the DMSO vehicle control to 9% with treatment of compound NSC96932 at a 1uM dose. Cell viability was reduced with treatment of compound NSC96932 compared to DMSO vehicle control around 50%. Data not shown.
